# Methylated Xanthones from the Rootlets of *Metaxya rostrata* Display Cytotoxic Activity in Colorectal Cancer Cells

**DOI:** 10.3390/molecules25194449

**Published:** 2020-09-28

**Authors:** Eva Mittermair, Hanspeter Kählig, Ammar Tahir, Stefanie Rindler, Xenia Hudec, Hemma Schueffl, Petra Heffeter, Brigitte Marian, Liselotte Krenn

**Affiliations:** 1 Department of Pharmacognosy, University of Vienna, Althanstraße 14, 1090 Vienna, Austria; ammar.tahir@univie.ac.at (A.T.); stefanie.rindler@gmail.com (S.R.); liselotte.krenn@univie.ac.at (L.K.); 2 Institute of Cancer Research, Department of Medicine I, and Comprehensive Cancer Center, Medical University Vienna, Borschkegasse 8a, 1090 Vienna, Austria; xenia.hudec@meduniwien.ac.at (X.H.); hemma.schueffl@meduniwien.ac.at (H.S.); petra.heffeter@meduniwien.ac.at (P.H.); brigitte.marian@meduniwien.ac.at (B.M.); 3 Department of Organic Chemistry, University of Vienna, Währingerstraße 38, 1090 Vienna, Austria; hanspeter.kaehlig@univie.ac.at

**Keywords:** *Metaxya rostrata*, metaxyaceae, 2-deprenyl-6-*O*-methyl-7-hydroxy-rheediaxanthone B, 2-deprenyl-5-*O*-methyl-7-methoxy-rheediaxanthone B, 2-deprenyl-5-*O*-methyl-7-hydroxy-rhee-diaxanthone B, 2-deprenyl-7-methoxy-rheediaxanthone B

## Abstract

The tree fern *Metaxya rostrata* (Kunth) C. Presl is common in the rainforests of Central and South America, where suspensions of the dried rhizome are traditionally used to treat intestinal diseases. Two compounds from this plant, 2-deprenyl-rheediaxanthone B (XB) and 2-deprenyl-7-hydroxy-rheediaxanthone B (OH-XB), have been shown to be biologically highly active against colorectal cancer (CRC) cells in previous studies. The current investigation resulted in the isolation of the previously undescribed methylated xanthones 2-deprenyl-6-*O*-methyl-7-hydroxy-rheediaxanthone B, 2-deprenyl-5-*O*-methyl-7-methoxy-rheediaxanthone B, 2-deprenyl-5-*O*-methyl- 7-hydroxy-rheediaxanthone B and 2-deprenyl-7-methoxy-rheediaxanthone B. All compounds were isolated by column chromatography, structures were elucidated by one- and two-dimensional NMR-experiments and the identities of the compounds were confirmed by LC-HRMS. In logarithmically growing SW480 CRC cell cultures, cytotoxicity by neutral red uptake and MTT assays as well as caspase activation was analyzed. Cellular targets were examined by Western blot, and topoisomerase I (topo I) inhibition potential was tested. Comparing the structure-activity relationship with XB and OH-XB, the monomethylated derivatives showed qualitatively similar effects/mechanisms to their nonmethylated analogues, while dimethylation almost abolished the activity. Inhibition of topo I was dependent on the presence of an unmethylated 7-OH group.

## 1. Introduction

*Metaxya rostrata* (Kunth) C. Presl was first described in 1836, and is the only species of the genus *Metaxya* that has been investigated phytochemically to date. The tree fern is widespread in lowland Amazonian forests, from Mexico to Panama, Peru, Bolivia and northern Brazil [1,2]. Especially in Costa Rica, its rhizome is used in ethnic medicine to treat intestinal diseases [3]. Considering the interest in natural compounds that mostly originates from traditional use and documentation, numerous valuable anticancer drugs have ultimately been obtained from plants [4,5,6,7]. Prenylated xanthones have gained a lot of interest during the last decade, as they are well known for their cytotoxic effects on malignant cells. The best studied among these are the mangostins that induce cell cycle arrest and active cell death in a broad range of tumor cells [8,9,10]. After establishing cytotoxic activity of fractions from an aqueous and a methanolic extract of *M. rostrata* against colorectal cancer (CRC) cells [3], an activity-guided study led to the isolation of structurally related 2-deprenyl-rheediaxanthone B (XB) and 2-deprenyl-7-hydroxy-rheediaxanthone B (OH-XB). These studies identified the two compounds as biologically highly active, blocking cell cycle progression and inducing cell death in CRC cells [3,11]. More recently, our findings suggested that both substances also interact with the cellular targets forkhead box protein M1 (FoxM1) and topoisomerase I (topo I) [12]. Thus, we observed that XB was a strong inducer of FoxM1 degradation, while OH-XB had a stronger impact on topo I activity. Based on these results, the focus of the current study was the isolation of further closely related xanthones in order to explore the efficacy and cellular targets in SW480 CRC cells in order to establish a detailed structure-activity relationship.

## 2. Results and Discussion

**Figure 1:**
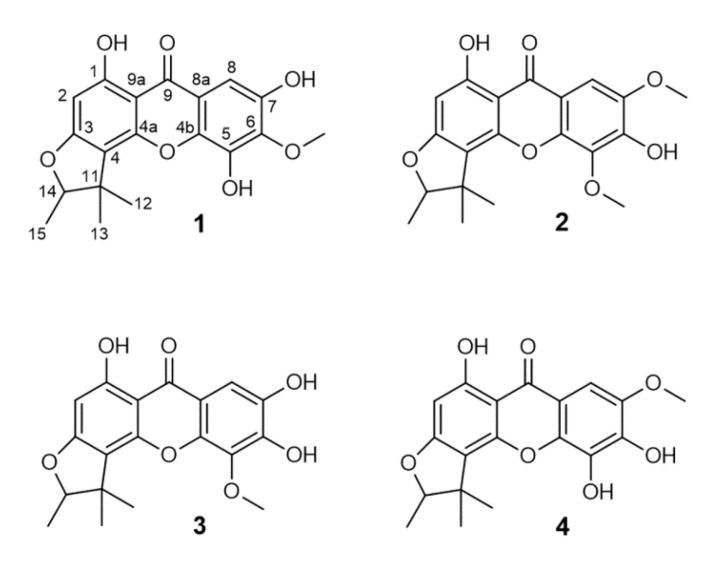
Chemical structures of the identified xanthones.

A detailed investigation of a CH_2_Cl_2_ extract of the dried rootlets from *M. rostrata* resulted in the isolation of three xanthones (**1**, **2**, **3**), and of an additional compound (**4**), which was only accessible as a mixture (**M**) together with **1** (Figure 1). They are all new, natural compounds and were identified by chromatographic, spectrometric and spectroscopic methods. This led to the elucidation of 2-deprenyl-6-*O*-methyl-7-hydroxy-rheediaxanthone B (**1**) as well as 2-deprenyl-5-*O*-methyl-7-methoxy-rheediaxanthone B (**2**). Compound **3** was identified as 2-deprenyl-5-*O*-methyl-7-hydroxy-rheediaxanthone B. In the mixture, together with **1**, compound **4** was identified as 2-deprenyl-7-methoxy-rheediaxanthone B at a ratio of 1:0.3, according to the integration in the ^1^H-NMR.

In general, the ^1^H- and ^13^C-NMR spectra are very similar to published data for 2-deprenyl-rheediaxanthone B [3]. For the prenyl part in all substances, the ^1^H-NMR spectra show two singlets at 1.33 and 1.62 ppm (two CH_3_), and one doublet at 1.41 ppm (CH_3_) coupled to a quartet at 4.54 ppm (CH), all within a shift range of ±0.015 ppm. In the aromatic region, proton 2 is detected around 6.13 ppm (±0.014 ppm). The second aromatic signal for H-8 is in the range from 7.08 to 7.36 ppm, influenced by the different methylation patterns. As only these two aromatic protons are present, the second aromatic ring has to carry three phenolic groups, i.e., either one being methylated or two in compound **2**. These methyl groups can be seen at around 3.95 ppm. In the ^13^C-NMR spectra, the signals for the prenyl part and the connected aromatic ring match very closely. To elucidate the substitution pattern, HMBC and NOESY spectra were recorded. In the HMBC spectra, the methyl protons give a three-bond correlation to the quaternary aromatic carbon, where the methoxy group is located. Methoxy substitution at position 6 or 7 shows a correlation to this carbon starting from H-8, either via three or two bonds, respectively. On the other hand, a methoxy group at position 7 gives a correlation in the NOESY spectrum to H-8, and in the case of a substitution at C-5, a minute cross peak can be seen in methyl groups 12 and 13 (Table 1, Table 2 and Table 3).

The identity, as determined by NMR, was further confirmed by LC-HRMS (see Section 3. General experimental procedures). Mixture **M** had a molecular formula of C_19_H_18_O_7_, as revealed by the ^13^C-NMR data and a protonated ion [M + H]^+^ at *m*/*z* 359.1138 (calcd. for C_19_H_19_O_7_, 359.1131) in the positive HRMS, proving that compounds **1** and **4** are regioisomeres. HRMS analyses revealed a quasimolecular ion [M + H]^+^ at *m*/*z* 359.1137 for compound **1** and at *m*/*z* 359.1132 for compound **3**, suggesting a molecular formula of C_19_H_18_O_7_ in conjunction with the ^13^C-NMR data (both calcd. for C_19_H_19_O_7_, 359.1131); thus, **3** is a third possible monomethylated regioisomer. For compound **2**, a molecular formula of C_20_H_20_O_7_ was assigned from its ^13^C-NMR data, and positive HRMS showed a quasimolecular ion [M + H]^+^ at *m*/*z* 373.1296 (calcd. for C_20_H_21_O_7_, 373.1290), confirming the additional methyl group.

**Figure 2:**
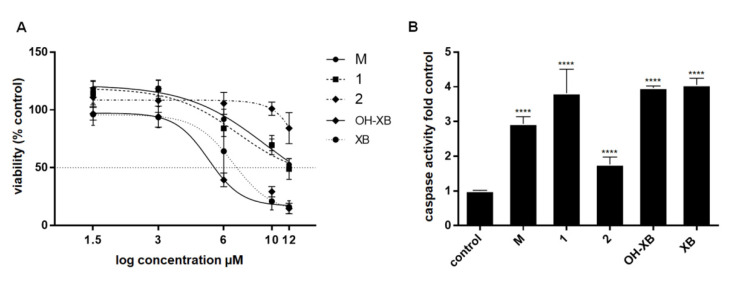
Cytotoxicity and caspase activity (**A**) Semiconfluent cultures of SW480 were exposed to the indicated concentrations of **M**, **1**, **2**, OH-XB and XB. Viability was determined after 48 h by neutral red uptake assay and MTT assay. The results are the mean ± SD pooled from four independent experiments (*n* = 4) performed in five-fold measurements. **M**, **1**, and **2** were less active than XB and OH-XB at *p* < 0.0001 according to two-way ANOVA of Data. (**B**) Protein lysates were harvested 48 h after exposure to 15 µM of **M**, **1**, **2**, OH-XB and XB and prepared to measure caspase activity. The results are the mean ± SD pooled from three independent experiments (*n* = 3) performed in duplicate. **** indicates a significant difference from control at *p* < 0.0001.

Due to sufficient purity, mixture **M** as well as compounds **1** and **2** could be evaluated in biological tests. For this purpose, **M**, **1** and **2**, as well as XB and OH-XB were dissolved in DMSO and diluted into the medium of semiconfluent SW480 CRC cells, which had shown the highest sensitivity to prenylated xanthones in our previous studies [3,12]. Cytotoxicity was determined by neutral red uptake and MTT assays after 48 h (Figure 2A). Based on the small amounts of compounds available and the consideration that a candidate drug should be effective at concentrations below 10 µM, 12 µM was the highest concentration tested for each compound in the viability tests. At that concentration, specific activity was 15% and 16% for OH-XB and XB respectively, while it was 49% and 54% for **1** and **M**, and 84% in cultures exposed to **2**, indicating that all three new compounds were less effective than XB and OH-XB. IC_50_ concentrations for the novel xanthones could not be determined, because the curves did not reach a bottom level. However, comparisons of the dose-response curves by two-way ANOVA demonstrated statistically significant differences between the methylated compounds and XB, as well as OH-XB, as shown in Table 4, that lists cytotoxic activity as defined by the extent of cell loss in the exposed culture. With this limitation, it was possible to draw two conclusions regarding the methylated compounds: (1) there were no differences between the effects of **M** and **1** at any concentration. As **M** consists of **1** with about 25% **4**, **4** and **1** seem to have similar activity. (2) Dimethylation in compound **2** strongly reduced the cytotoxic effect.

Induction of apoptosis was tested by induction of caspase activity using a standard enzyme assay with the substrate Ac-DEVD-APC, as shown in our previous studies [11,12]. After exposure to 15 µM of **M**, **1**, **2**, OH-XB and XB for 48 h, we found a significant activation of caspase compared to control (Figure 2B). All prenylated xanthones induced caspase activity with **M** and **1** reaching similar levels as XB and OH-XB. Activation by compound **2** was significantly less compared to those by the other compounds, in line with the difference in cytotoxic activity. This indicates that the mode of action of the compounds involves the activation of caspase-dependent cell death.

Cytotoxic activity and induction of active cell death is a common effect of plant-derived xanthones [13]. The best studied compound in this category is α-mangostin, that has been shown to induce cell cycle arrest and apoptosis in several malignant cell lines [10,14,15]. Our own previous studies described similar effects for XB and OH-XB [3,11,12]. To evaluate the cellular mechanisms triggered by the new xanthones, cellular targets established in previous work [3,11,12] were studied. Specifically, FoxM1, as well as the mitotic cyclins A and B [16], were analyzed by western blotting.

Loss of the cellular target FoxM1 was identified as a common effect of OH-XB and XB [11]. This effect was obtained by a combination of inhibited gene expression on the mRNA level and active degradation of the protein [12]. The FoxM1 transcription factor is essential for the execution of mitosis, upregulated in many malignancies, and plays a key role in oncogenesis [17,18,19,20]. The new methylated compounds reduced FoxM1 levels after 48 h of treatment. This effect was not only observed with **M** and **1,** but also with **2**. This is the only strong effect induced by **2**, and needs further analysis, as it may be related to alterations in the malignant characteristics of the treated cells [17]. The xanthone mangiferin has been reported to modulate epithelial-mesenchymal transition [21], an effect that may well be mediated by FoxM1 [22]. However, for this purpose, new plant material has to be extracted, as in this study, the required amounts of compounds **1** and **2** were not available for further functional assays.

**Figure 3:**
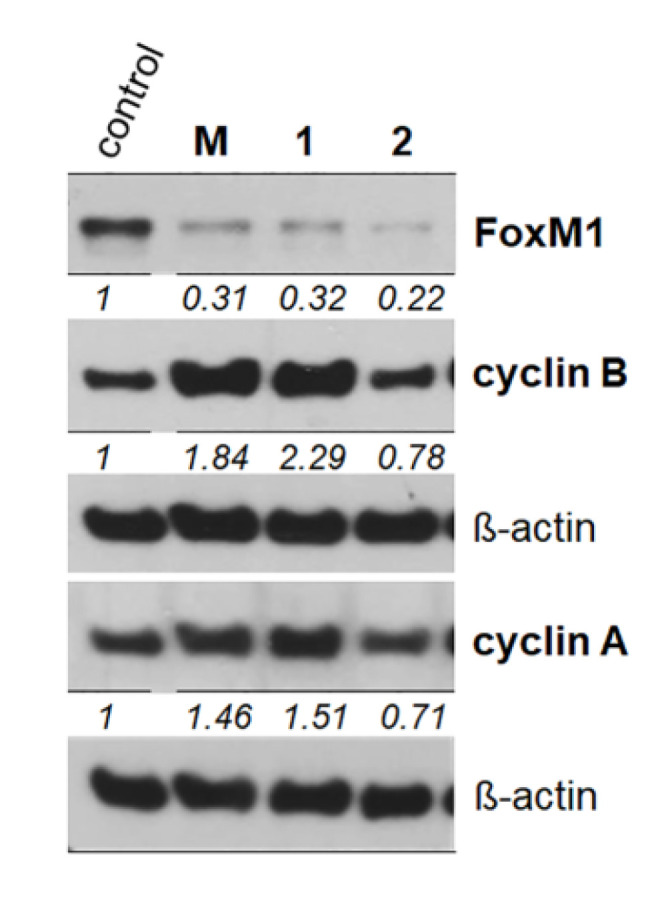
Cell cycle-related proteins. Protein lysates of SW480 cells were harvested after 48 h of exposure to 15 µM **M**, **1** and **2**, and the protein levels of FoxM1, cyclin B and cyclin A were analyzed by western blotting. The numbers below the bands give the mean fold increase of three independent experiments (*n* = 3, control set to ß-actin).

Cyclins A and B are the main mitotic cyclins and are both required for viability, as shown by experiments with knockout mice [16]. Determination of their protein level was used as a compound-sparing way of cell cycle analysis (Figure 3). Cyclin B was significantly upregulated with **M** and **1**, indicating that cells accumulate in the G2/M-phase after treatment. The results for cyclin A, an indicator for early mitotic events, were similar but less pronounced: protein abundance was increased by about 50% after treatment with **M** and **1**. In our recent analysis of XB and OH-XB, a comparable increase of cyclins A and B was only observed in XB-exposed cultures; this was correlated with cell cycle arrest in G2/M. After treatment with OH-XB, cyclin A was downregulated and cyclin B strongly decreased, which was also reflected by a cell cycle arrest in the S-phase but not in G2/M [11]. In cells exposed to compound **2**, neither an increase nor a decrease in the mitotic cyclins was observed, suggesting that cell cycle distribution was not altered. This is in line with the known low cytotoxic activity of the dimethylated xanthone.

**Figure 4:**
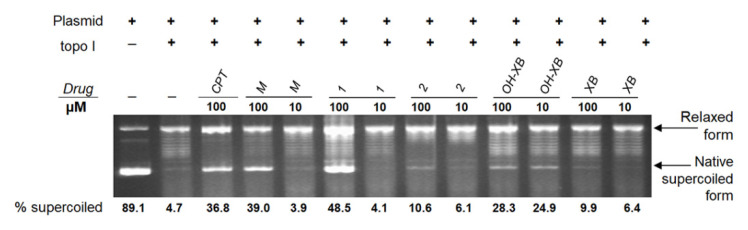
Inhibition of topoisomerase I. First, 250 ng plasmid DNA (pGEM1) was incubated with 10 µM and 100 µM of **M**, **1**, **2**, OH-XB and XB containing 2 µL topo I. The percentage of supercoiled DNA was then determined by the ratio of supercoiled to relaxed DNA. The numbers below the bands give the mean fold increase of three independent experiments (*n* = 3).

The second cellular activity that has been previously described specifically for OH-XB was the inhibition of topo I [12]. Therefore, we evaluated the impact of **M**, **1** and **2** on topo I activity in comparison to the positive control camptothecin (CPT), as well as to XB and OH-XB (Figure 4), by a DNA relaxation assay. Topo I inhibition by XB and the dimethylated compound **2** showed a low amount of restored supercoiled DNA. In contrast, mixture **M** and compound **1** produced a drastic inhibitory effect after treatment at a concentration of 100 µM. However, no inhibition was detected at 10 µM. Only OH-XB distinctly inhibited topo I at both concentrations, i.e., 10 and 100 µM.

## 3. Materials and Methods

### 3.1. General Experimental Procedures

Column chromatography was performed on Sephadex LH-20 under elution with MeOH 50% to 80% (column diameter and height: 1 cm and 30 cm). For TLC silica gel plates (Merck, Darmstadt, Germany) and the mobile phases (1) toluene-ethyl formate-HCOOH (10:4:1); (2) EtOAc-HCOOH-MeOH-H_2_O (70:8:8:11); (3) EtOAc-HCOOH-MeOH-H_2_O (70:8:4:4) were used; detection: UV_254nm_; anisaldehyde-H_2_SO_4_-reagent; Naturstoff-reagent A/PEG 400 under UV_366nm_.

For LC-HRMS analyses, the Ultimate 3000 UHPLC system (Thermo Fisher Scientific, San Jose, CA, USA) was equipped with a reversed-phase C18 column (Kinetex; 2.1 mm × 15 cm, 2.6 μm, C18 100 Å). Mobile phases were A) H_2_O-HCOOH (100:0.02) (*v*/*v*) and B) ACN-MeOH-HCOOH (80:20:0.02) (*v*/*v*). A 10 min binary gradient with flow rate set to 250 μL/min was applied as follows: 0−1 min, 5% B; 2−6 min, 5−85% B; 6–8 min, 95% B; 8–10 min re-equilibration with 5% B). 5 µL of each sample (1 mg/mL) were injected. Mass spectrometric detection was performed with an ESI-Qq-TOF mass spectrometer (micrOTOF-Q II, maXis HD, Bruker Compass, Bremen, Germany), 200 °C heater temperature, 4 L/min drying gas and 0.4 bar nebulizer gas. 2.0 KV spray voltage at 250 °C capillary temperature were applied to achieve negative/positive ion mode ionization. MS1 scans were performed with an *m*/*z* range from 150 to 1500. MS/MS scans of the most abundant ions were achieved through high collisional dissociation (HCD) fragmentation at 55.0 eV normalized collision energy.

All NMR spectra were recorded on a Bruker Avance III HD 700 NMR spectrometer (Bruker BioSpin, Rheinstetten, Germany) using a 5 mm helium cooled cryo probe (QCI-F) with z axis gradients and automatic tuning and matching accessory. The resonance frequency for ^1^H-NMR was 700.40 MHz, for ^13^C-NMR 176.12 MHz. All measurements were performed for a solution in fully deuterated methanol at a temperature of 298 K. Standard 1D, like ^13^C-DEPTQ, and gradient-enhanced (ge) 2D experiments, like double quantum filtered (DQF) COSY, TOCSY, NOESY (mixing time 800 ms), HSQC, and HMBC (long range J coupling 8 Hz), were used as supplied by the manufacturer. Chemical shifts are referenced internally to the residual, nondeuterated solvent signal for ^1^H (δ = 3.31 ppm) or to the carbon signal of the solvent for ^13^C (δ = 49.00 ppm).

### 3.2. Chemicals and Reagents

All solvents were analytical-grade or HPLC-grade. ACN (Chromanorm), EtOAc (Normapur) and MeOH (LiChrosolv) were obtained from VWR International (Radnor, Pennsylvania, PA, USA). Toluene and ethylformate were acquired from Merck KGaA (Darmstadt, Germany). HCOOH was obtained from Carl Roth GmbH (Karlsruhe, Germany).

### 3.3. Plant Material

Underground parts of *Metaxya rostrata* were collected in February 2003 in La Gamba, Costa Rica and authenticated in the Herbarium of the Museo National in San Jose by Dr. W. Huber (University of Vienna). Voucher specimens are deposited under the number MR0203 at the Herbarium of the Department of Pharmacognosy, University of Vienna, Austria.

### 3.4. Extraction and Isolation

The dried rootlets of *M. rostrata* (920 g) were extracted by sonification with CH_2_Cl_2_, resulting in 5.3 g CH_2_Cl_2_ extract which was separated by column chromatography on Sephadex LH-20 under elution with EtOAc. Two hundred and ninety fractions were pooled to obtain 16 subfractions (CC-13/1 to CC-13/16) [23]. The most active subfractions were eluted sequentially by solid phase extraction (SPE) using four reservoir volumes of 60%, 70%, 80%, 90% and 100% MeOH [3,23]. Fractions CC-13/9 to CC-13/11 were pooled and eluted with 80% MeOH, resulting in fraction SPE-9-11/80%. Fraction CC-13/6 was eluted with 80% MeOH resulting in fraction SPE-6/80% and fraction CC-13/7 was eluted with 60% MeOH, resulting in fraction SPE-13/60%.

Fraction SPE-9-11/80% (10.2 mg), as well as SPE-13/60% (5.7 mg) and SPE-6/80% (7.7 mg), were further analyzed by TLC and LC-MS. SPE-9-11/80% was subjected to column chromatography (CC1) on Sephadex LH-20 (1 × 30 cm; 2 mL/10 min; eluent 50% MeOH) to obtain 11 subfractions, resulting in the isolation of mixture M (8 mg, purity > 95%), at a ratio of 1:0.3. Fractionation of SPE-13/60% by CC2 on Sephadex LH-20 (1 × 30 cm; 1 mL/10 min; eluent 50 to 80% MeOH; 14 subfractions) yielded compounds 1 (2.85 mg, purity > 98%) and 3 (1.28 mg, purity > 50%). Compound 2 (0.7 mg; purity > 96%) was obtained from fraction SPE-6/80% in the same way, by CC3 (Sephadex LH-20; 1 × 30 cm; 1 mL/10 min; eluent 50% MeOH; 10 subfractions).

*2-Deprenyl-6-O-methyl-7-hydroxy-rheediaxanthone B* (**1**)*, 2-deprenyl-7-methoxy-rheediaxanthone B* (**4**) *[ratio 1:0.3]* (**M**), yellow needles, ^1^H-NMR (700.40 MHz, CD_3_OD) and ^13^C-NMR (176.12 MHz, CD_3_OD) data: see Table 1; HRMS *m*/*z* 359.1138 [M + H]^+^ (calcd. for C_19_H_19_O_7_, 359.1131); see also Supplementary Figures S1–S8.

*2-Deprenyl-6-O-methyl-7-hydroxy-rheediaxanthone B* (**1**), yellow microcrystalline powder, ^1^H-NMR (700.40 MHz, CD_3_OD) and ^13^C-NMR (176.12 MHz, CD_3_OD) data: see Table 1; HRMS *m*/*z* 359.1137 [M + H]^+^ (calcd. for C_19_H_19_O_7_, 359.1131); see also Supplementary Figures S9–S16.

*2-Deprenyl-5-O-methyl-7-methoxy-rheediaxanthone B* (**2**), yellowish amorphous powder, ^1^H-NMR (700.40 MHz, CD_3_OD) and ^13^C-NMR (176.12 MHz, CD_3_OD) data: see Table 2; HRMS *m*/*z* 373.1296 [M + H]^+^ (calcd. for C_20_H_21_O_7_, 373.1290); see also Supplementary Figures S17–S24.

*2-Deprenyl-5-O-methyl-7-hydroxy-rheediaxanthone B* (**3**), pale yellowish amorphous powder, ^1^H-NMR (700.40 MHz, CD_3_OD) and ^13^C-NMR (176.12 MHz, CD_3_OD) data: see Table 2; HRMS *m*/*z* 359.1132 [M + H]^+^ (calcd. for C_19_H_19_O_7_, 359.1131); see also Supplementary Figures S25–S31.

### 3.5. Cell Lines

SW480 CRC cells were obtained from ATCC and kept under standard tissue culture conditions (5% CO_2_ at 37 °C) using minimal essential medium (MEM) containing 10% fetal calf serum (FCS). Cells were regularly checked for *Mycoplasma* contamination.

### 3.6. Cytotoxic Activity

SW480 cells were plated in 96-well plates at 1 × 10^4^/well and left to attach for 24 h; semiconfluent cultures were exposed to **M**, **1**, **2**, OH-XB and XB at increasing concentrations (1.5–12 µM) for 48h. Stock solutions of the compounds were prepared in DMSO and stored at −20 °C. Control media contained the appropriate volume of DMSO with a maximum of 0.12% in the final solution. Cell viability was determined by neutral red uptake assay or MTT assay as indicated [3,11] and measured at the Tecan infinite 200Pro. Cytotoxic activity was calculated as loss of viability (100-viability).

### 3.7. Caspase Activity

Cells were washed, homogenized in lysis buffer [50 mM Tris/HCl (pH 7.4), 500 mM NaCl, 1% NP-40, 0.5% Na-DOC, 0.1% SDS, 0.05% NaN_3_, 10 mM NaF, 500 mM *o*-vanadate] and supplemented with the protease inhibitor complete [3]. Assessment of caspase activity was performed as reported in [24].

### 3.8. Western Blot

Cells were homogenized using lysis buffer supplemented with complete to block proteolytic enzymes, as well as sodium fluoride and sodium vanadate to inhibit phosphatases [3,11]. Then, 20 µg of protein was analyzed by electrophoresis on 12% polyacrylamide gels and transferred to PVDF blotting membranes (GE Healthcare, Chicago, IL, USA). Primary antibodies used targeted FoxM1 (#5436, Cell Signaling Technology (CST), Danvers, MA, USA), cyclin A (#1109, Santa Cruz Biotechnology, Dallas, TX, USA), cyclin B (#4138, CST) and ß-actin (A5441, Sigma-Aldrich, St. Louis, MO, USA). Detection was achieved by HRP-linked secondary antibodies and ECL^TM^ Prime Western Blotting Detection Reagent (RPN 2236, GE Healthcare) [11].

### 3.9. Topoisomerase I Assay

Topo I activity was investigated as relaxation of supercoiled plasmid DNA (pGEM1) isolated from *E. coli*. Therefore, 250 ng of plasmid DNA were incubated for 30 min at 37 °C with 10 µM and 100 µM of the test compounds (dissolved in DMSO) in a final volume of 30 µL, containing 2 µL topo I-containing nucleic extract isolated from MCF-7 cells [25], 10 mM Tris (pH 7.9), 100mM KCl, 10 mM MgCl_2_, 0.5 mM dithiothreitol, 0.5 mM ethylenediaminetetraacetic acid, and 0.03 mg/mL bovine serum albumin (BSA). As a positive control, 100 µM camptothecin (CPT) (#11694, Cayman chemical) was used. The reaction was stopped by incubation with 6 µL 5% sodium dodecyl sulfate containing 1 mg/mL proteinase K at 43 °C for 30 min. Samples were separated by submarine 1% agarose gel electrophoresis (55 V, 2 h); gels were stained with 10 µL/100 mL ethidium bromide for 20 min. UV-transilluminated gels (Gel Doc^TM^ XR, BioRad) were documented by Quantity One analysis software (version 4.6).

### 3.10. Statistical Analysis

Data were calculated with analysis of variance (ANOVA), two-way ANOVA or Student’s *t* test as appropriate using Graph Pad Prism 6.01 and Image J-win64 software. Significance was designated as * for *p* < 0.05, ** for *p* < 0.01, *** for *p* < 0.001, and **** for *p* < 0.0001. Error bars depict ± SD for *n* = 3 or *n* = 4.

## 4. Conclusions

A phytochemical approach led to the isolation of methylated xanthones from the underground parts of *Metaxya rostrata* (**M**, **1**, **2** and **3**). The structures were elucidated based on thorough 1D and 2D NMR, as well as LC-HRMS measurements. Looking at structure activity relationships, the monomethylated xanthones induced cell loss, activation of caspase and cell cycle arrest in G2/M in CRC cells, indicating that the compounds affected cell proliferation and induced their apoptotic cell death. Interestingly, their cytotoxic effects were reduced compared to those of the unmethylated compounds XB and OH-XB. In addition, dimethylation in compound **2** drastically reduced the activity, suggesting that the presence of two methyl groups disrupted the interactions with crucial cellular targets. The target responsible for the cytotoxic activity of these xanthones is unlikely to be the transcription factor FoxM1, because all compounds, including **2**, suppressed FoxM1.

The inhibition of topo I correlated with an unmethylated OH-group at position 7, as shown by the high activity of OH-XB versus XB [12], and by the significant reduction when this OH-group was methylated in compound **2**. Compound **1**, with its unmethylated OH-group in position 7, displayed considerable inhibition of topo I, i.e., comparable to that of OH-XB. Unfortunately, we cannot draw any conclusions about **4**, which also carries a methyl group at the 7 OH-group, because it was only available in a mixture with **1**. In summary, our observations showed cytotoxic activity of the new xanthones **M** and **1** for the first time. As topo I inhibitors are highly effective cancer therapeutics [26,27,28], this defines a promising lead structure for further preclinical investigations using models of CRC, but also other malignancies.

## Figures and Tables

**Table 1:** ^1^H- and ^13^C-NMR data of mixture **M** in CD_3_OD.

Mixture M Compound 1					Mixture M Compound 4				
Atom Position		^1^H (ppm)	*J*_H.H_ (Hz)	^13^C (ppm)	Atom Position		^1^H (ppm)	*J*_H.H_ (Hz)	^13^C (ppm)
1	C	-	-	165.13	1	C	-	-	164.96
2	CH	6.120	s	93.99	2	CH	6.118	s	93.98
3	C	-	-	167.27	3	C	-	-	166.94
4	C	-	-	114.12	4	C	-	-	114.13
4a	C	-	-	154.44	4a	C	-	-	154.30
4b	C	-	-	148.90	4b	C	-	-	e ^1^ 143.66
5	C	-	-	140.89	5	C	-	-	134.94
6	C OCH_3_	- 3.966	s	143.47 61.19	6	C	-	s	e 143.69
7	C	-	-	141.83	7	C OCH_3_	- 3.948	s	147.27 56.58
8	CH	7.083	s	100.46	8	CH	7.170	s	96.47
8a	C	-	-	117.60	8a	C		-	112.93
9	C	-	-	181.70	9	C	-	-	181.50
9a	C	-	-	104.28	9a	C	-	-	104.08
11	C	-	-	44.95	11	C	-	-	44.98
12	CH_3_	1.604	s	25.92	12	CH_3_	1.611	s	25.95
13	CH_3_	1.316	s	21.39	13	CH_3_	1.320	s	21.42
14	CH	4.530	q 6.6	92.28	14	CH	4.525	q 6.6	92.21
15	CH_3_	1.398	q 6.6	14.56	15	CH_3_	1.399	q 6.6	14.56

^1^ Assignment exchangeable.

**Table 2:** ^1^H- and ^13^C-NMR data of compound **1** and **2** in CD_3_OD.

Compound 1					Compound 2				
Atom Position		^1^H (ppm)	*J*_H.H_ (Hz)	^13^C (ppm)	Atom Position		^1^H (ppm)	*J*_H.H_ (Hz)	^13^C (ppm)
1	C	-	-	165.16	1	C	-	-	164.98
2	CH	6.139	s	93.99	2	CH	6.145	s	94.20
3	C	-	-	167.30	3	C	-	-	166.92
4	C	-	-	114.14	4	C	-	-	114.10
4a	C	-	-	154.47	4a	C	-	-	154.08
4b	C	-	-	148.94	4b	C	-	-	147.98
5	C	-	-	141.00	5	C OCH_3_	- 3.969	- s	136.75 61.76
6	C OCH_3_	- 3.967	- s	143.52 61.18	6	C	-	-	151.97
7	C	-	-	141.89	7	C OCH_3_	- 3.944	- s	148.76 56.59
8	CH	7.090	s	100.39	8	CH	7.359	s	100.67
8a	C	-	-	117.62	8a	C	-	-	111.67
9	C	-	-	181.75	9	C	-	-	180.93
9a	C	-	-	104.30	9a	C	-	-	104.07
11	C	-	-	44.97	11	C	-	-	44.97
12	CH_3_	1.613	s	25.95	12	CH_3_	1.626	s	26.12
13	CH_3_	1.327	s	21.39	13	CH_3_	1.338	s	21.72
14	CH	4.547	q 6.6	92.31	14	CH	4.555	q 6.6	92.13
15	CH_3_	1.407	d 6.6	14.58	15	CH_3_	1.417	d 6.6	14.53

**Table 3:** ^1^H- and ^13^C-NMR data of compound **3** in CD_3_OD.

Compound 3				
Atom Position		^1^H (ppm)	*J*_H.H_ (Hz)	^13^C (ppm)
1	C	-	-	164.95
2	CH	6.117	s	93.99
3	C	-	-	166.74
4	C	-	-	113.93
4a	C	-	-	154.11
4b	C	-	-	148.34
5	C OCH_3_	- 3.954	- s	136.60 61.78
6	C	-	-	u ^1^ 139.09
7	C	-	-	u 141.84
8	CH	7.224	s, br	102.75
8a	C	-	-	u 114.79
9	C	-	-	181.23
9a	C	-	-	104.05
11	C	-	-	44.96
12	CH_3_	1.624	s	26.14
13	CH_3_	1.332	s	21.72
14	CH	4.536	q 6.6	92.05
15	CH_3_	1.410	d 6.6	14.52

^1^ assignment uncertain.

**Table 4:** Sensitivity of SW480 cells to **M**, **1**, **2**, OH-XB and XB.

	% Cytotoxic Activity (12 µM)	SD
**M**	46.04	±3.34
**1**	51.04	±6.00
**2**	15.79	±13.45
OH-XB	85.32	±4.53
XB	83.99	±5.44

## Supplementary Materials

The following are available online, Figure S1: ^1^H-NMR spectrum of mixture **M** (CD_3_OD, 700.40 MHz), Figure S2: ^13^C-NMR spectrum of mixture **M** (CD_3_OD, 176.12 MHz), Figure S3: HSQC spectrum of mixture **M** (CD_3_OD), Figure S4: HMBC spectrum of mixture **M** (CD_3_OD), Figure S5: NOESY spectrum of mixture **M** (CD_3_OD), Figure S6: MS1 spectrum of mixture **M** showing the adduct [M + H]^+^, Figure S7: MS1 spectrum of mixture **M** showing the adduct [M − H]^−^, Figure S8: MS2 spectrum of mixture **M** fragmented using HCD at 55.0 eV, Figure S9: ^1^H-NMR spectrum of compound **1** (CD_3_OD, 700.40 MHz), Figure S10: ^13^C-NMR spectrum of compound **1** (CD_3_OD, 176.12 MHz), Figure S11: HSQC spectrum of compound **1** (CD_3_OD), Figure S12: HMBC spectrum of compound **1** (CD_3_OD), Figure S13: NOESY spectrum of compound **1** (CD_3_OD), Figure S14: MS1 spectrum of compound **1** showing the adduct [M + H]^+^, Figure S15: MS1 spectrum of compound **1** showing the adduct [M − H]^−^, Figure S16: MS2 spectrum of compound **1** fragmented using HCD at 55.0 eV, Figure S17: ^1^H-NMR spectrum of compound **2** (CD_3_OD, 700.40 MHz), Figure S18: ^13^C-NMR spectrum of compound **2** (CD_3_OD, 176.12 MHz), Figure S19: HSQC spectrum of compound **2** (CD_3_OD), Figure S20: HMBC spectrum of compound **2** (CD_3_OD), Figure S21: NOESY spectrum of compound **2** (CD_3_OD), Figure S22: MS1 spectrum of compound **2** showing the adduct [M + H]^+^, Figure S23: MS1 spectrum of compound **2** showing the adduct [M − H]^−^, Figure S24: MS2 spectrum of compound **2** fragmented using HCD at 55.0 eV, Figure S25: ^1^H-NMR spectrum of compound **3** (CD_3_OD, 700.40 MHz), Figure S26: HSQC spectrum of compound **3** (CD_3_OD), Figure S27: HMBC spectrum of compound **3** (CD_3_OD), Figure S28: NOESY spectrum of compound **3** (CD_3_OD), Figure S29: MS1 spectrum of compound **3** showing the adduct [M + H]^+^, Figure S30: MS1 spectrum of compound **3** showing the adduct [M − H]^−^, Figure S31: MS2 spectrum of compound **3** fragmented using HCD at 55.0 eV.
