# Supplementary material for: Methylated Xanthones from the Rootlets of Metaxya rostrata Display Cytotoxic Activity in Colorectal Cancer Cells
Source: Molecules. 2020 Sep 28;25(19):4449. doi: 10.3390/molecules25194449 (PMC7582535; doi:10.3390/molecules25194449)
Supplement: Supplementary file 1 [file molecules-25-04449-s001.pdf]

Supplementary material

Supplemental methods

All NMR spectra were recorded on a Bruker Avance III HD 700 NMR spectrometer (Bruker BioSpin, Rheinstetten, Germany) using a 5 mm helium cooled cryo probe (QCI-F) with z axis gradients and automatic tuning and matching accessory. The resonance frequency for ^{1}H NMR was 700.40 MHz, for ^{13}C NMR 176.12 MHz.

LC-HRMS analyses were carried out on an Ultimate 3000 UHPLC system (Thermo Fisher Scientific - San Jose, CA) coupled to a ESI-Qq-TOF mass spectrometer (micrOTOF-Q II, Bruker Compass, maXis HD). MS1 scans were performed with an m/z range from 150 to 1500. MS2 scans of the most abundant ion were achieved through High collisional dissociation (HCD) fragmentation at 55.0 eV normalized collision energy.

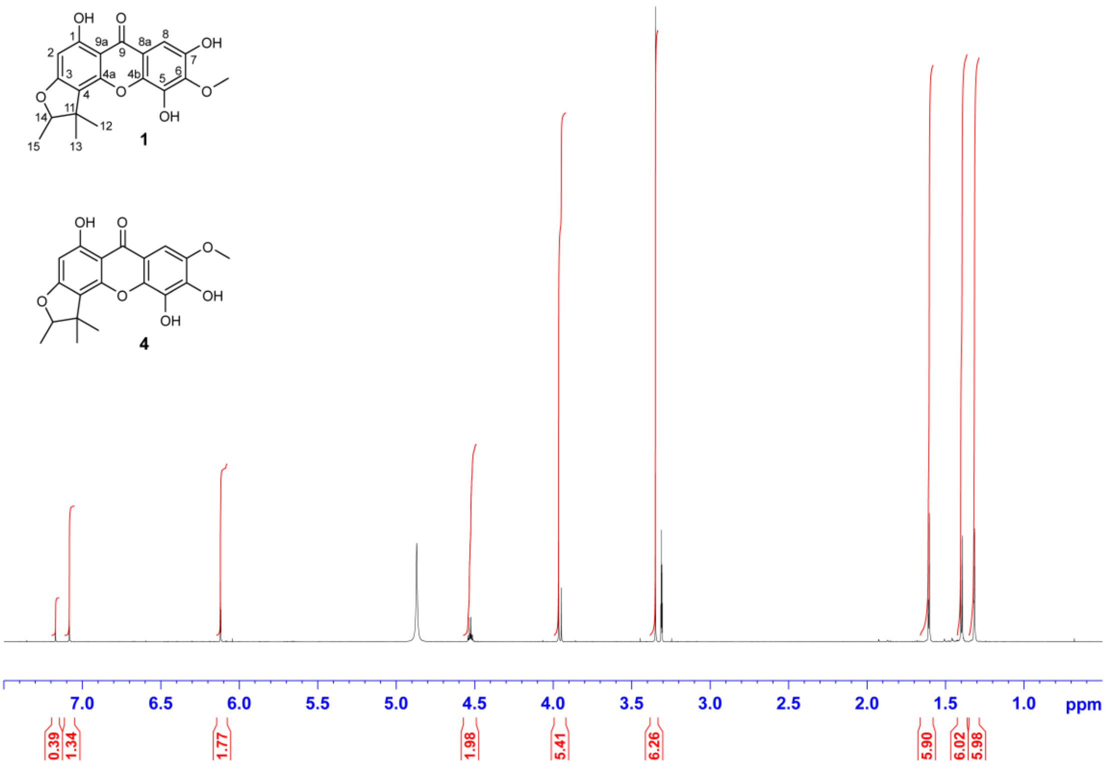

Fig.S1: ^{1}H NMR spectrum of mixture **M** (CD_3OD, 700.40 MHz)

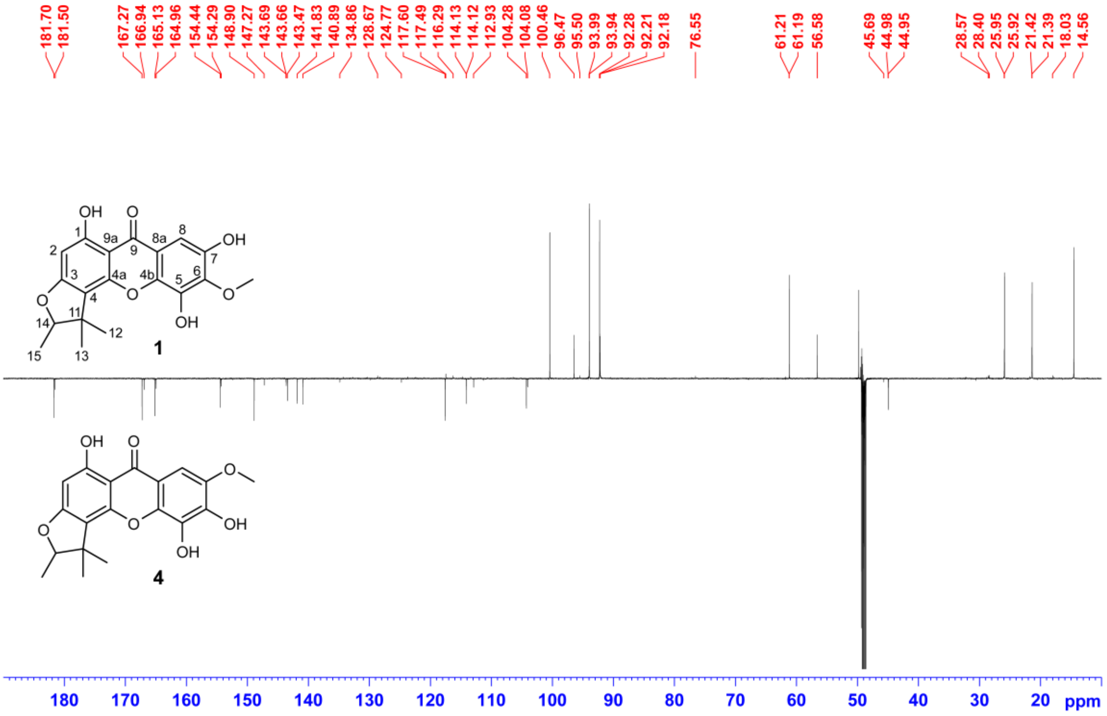

Fig.S2: ^{13}C NMR spectrum of mixture **M** (CD_3OD, 176.12 MHz)

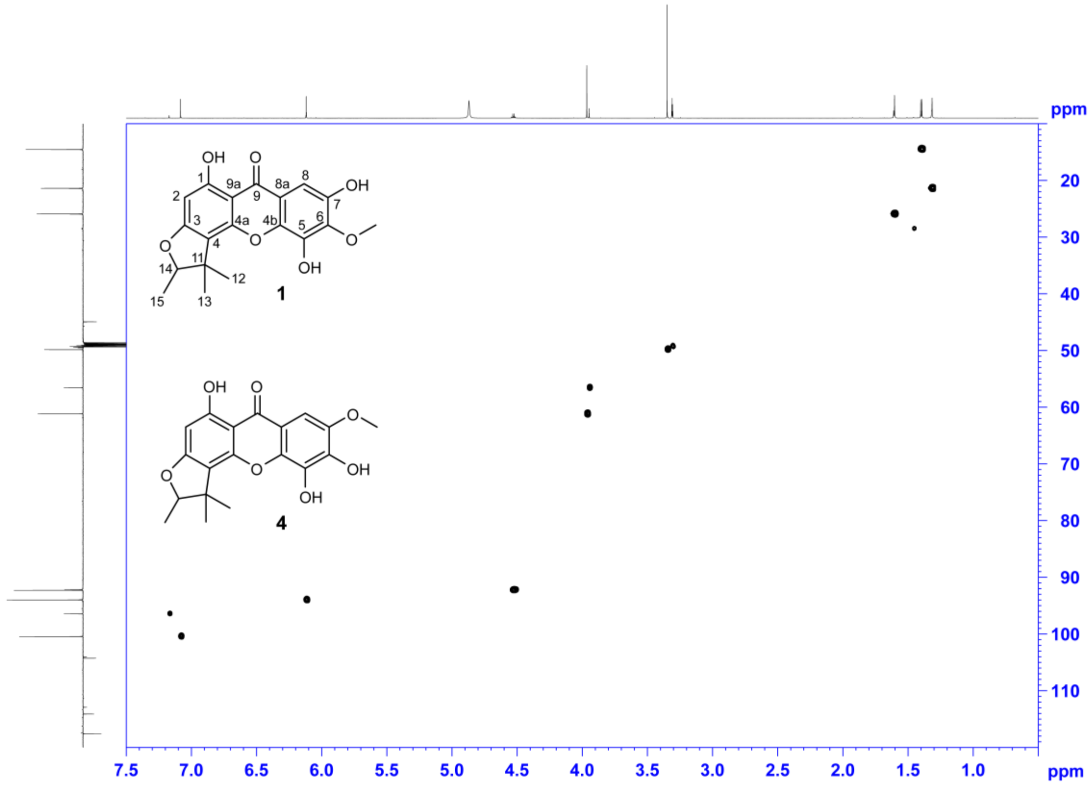

Fig.S3: HSQC spectrum of mixture **M** (CD_3OD)

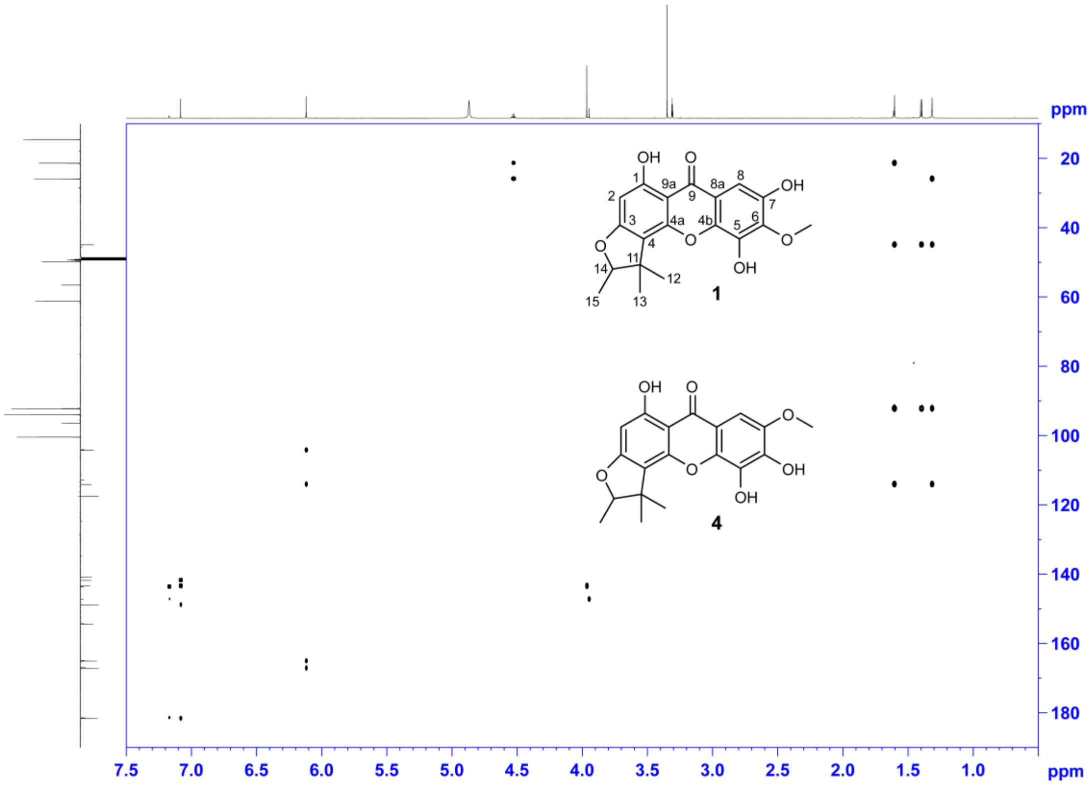

Fig.S4: HMBC spectrum of mixture **M** (CD_3OD)

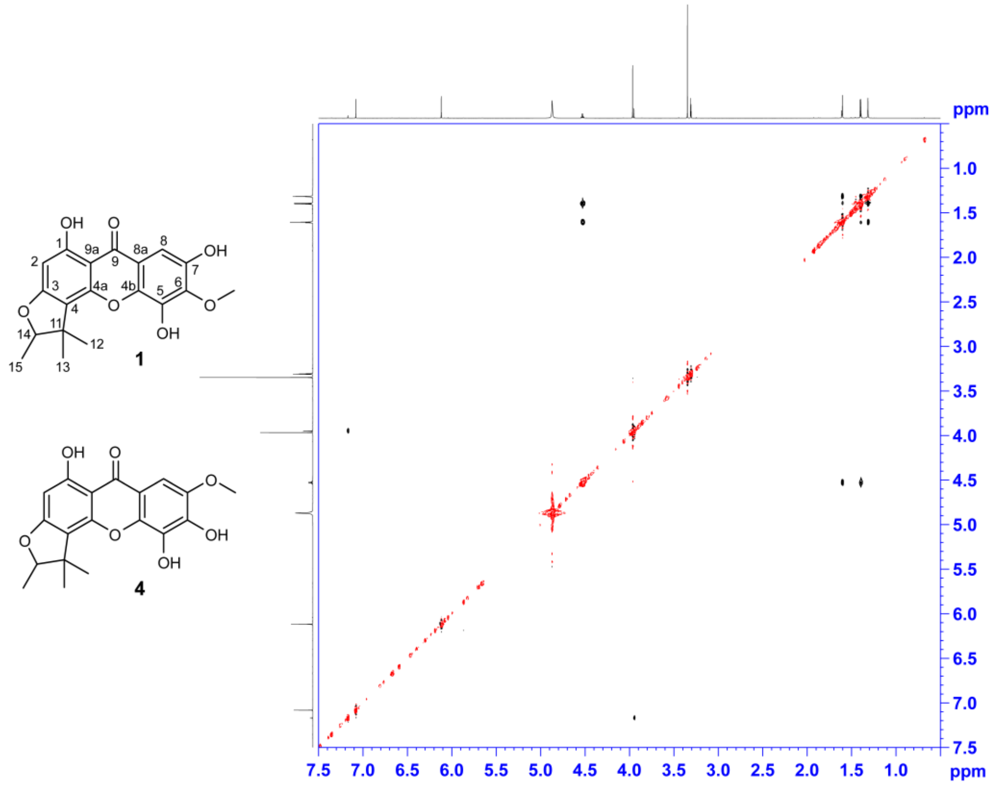

Fig.S5: NOESY spectrum of mixture **M** (CD_3OD)

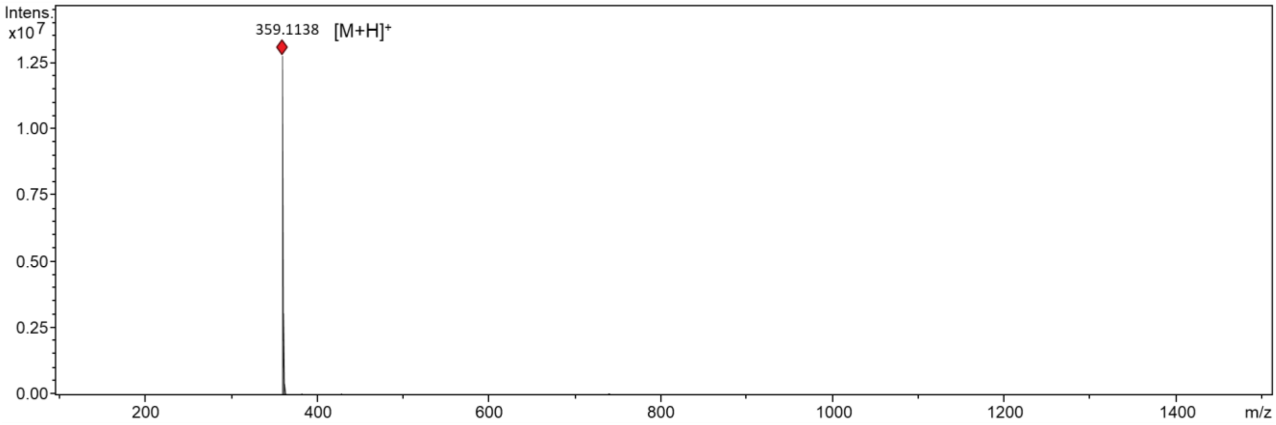

Fig.S6: MS1 spectrum of mixture **M** showing the adduct $[M+H]^{+}$

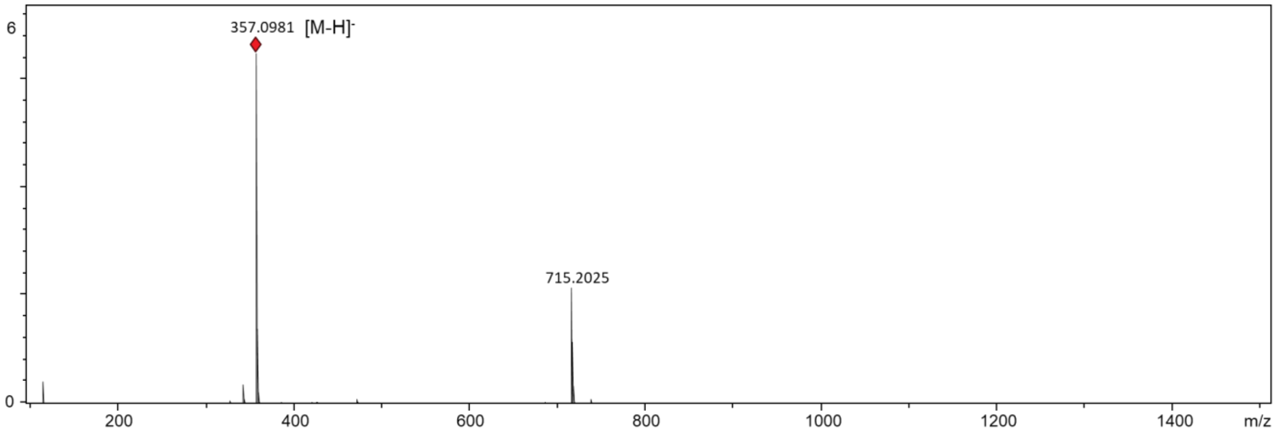

Fig.S7: MS1 spectrum of mixture **M** showing the adduct $[M-H]^{-}$

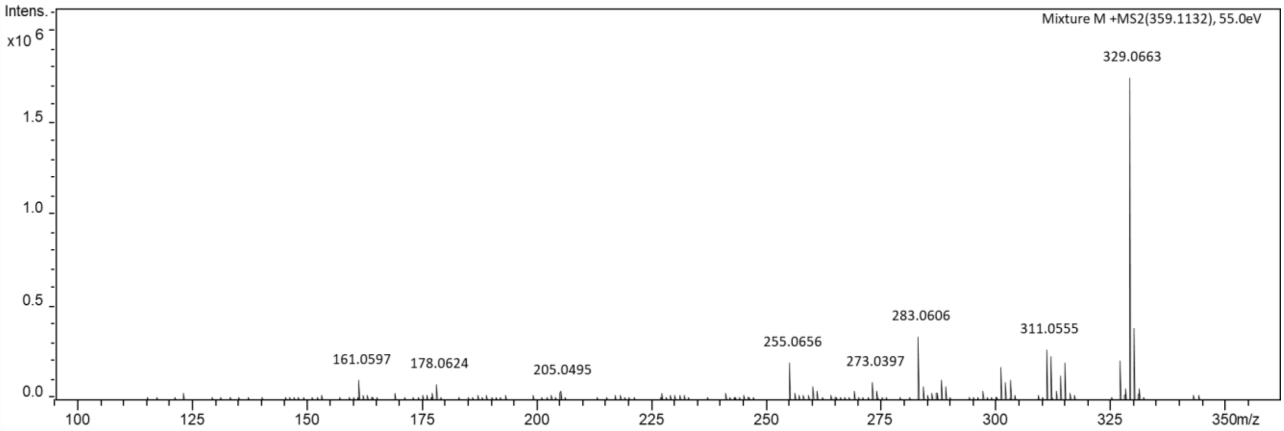

Fig.S8: MS2 spectrum of mixture **M** fragmented using HCD at 55.0eV

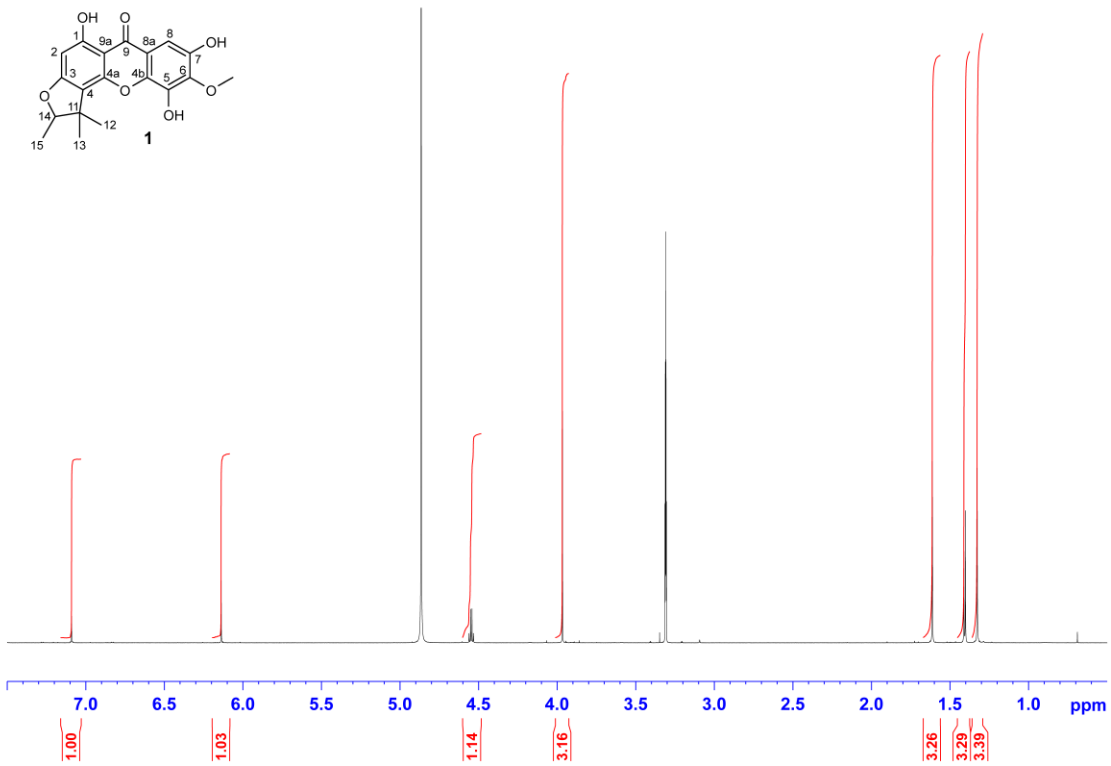

Fig.S9: ^{1}H NMR spectrum of compound **1** (CD_3OD, 700.40 MHz)

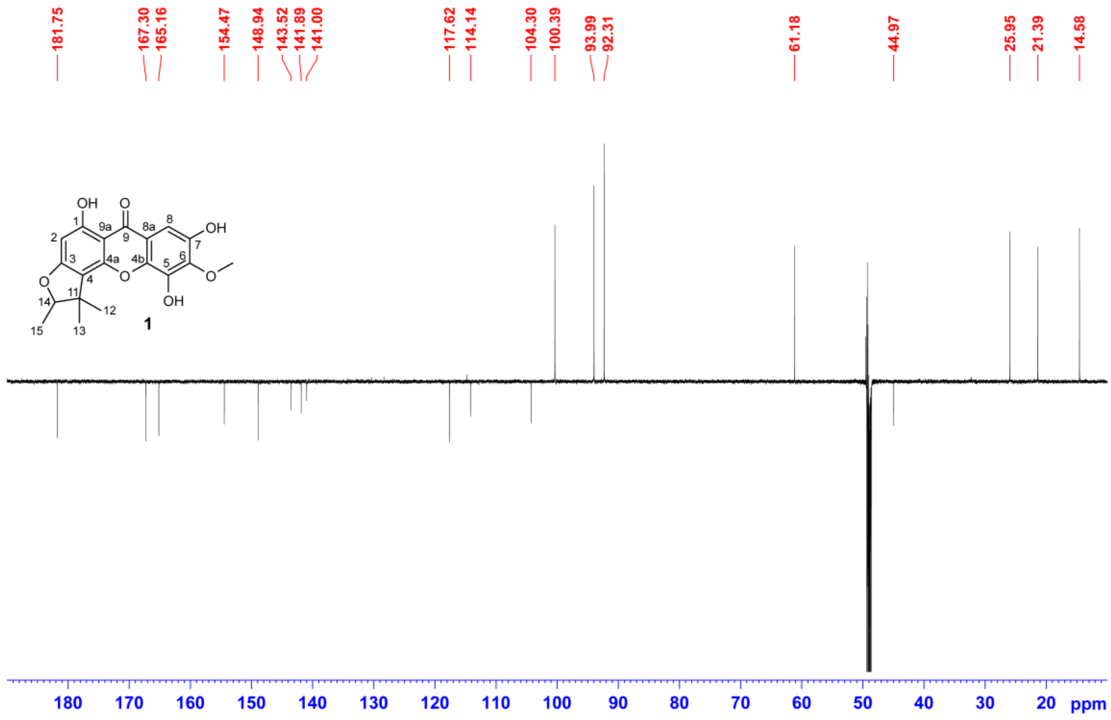

Fig.S10: ^{13}C NMR spectrum of compound **1** (CD_3OD, 176.12 MHz)

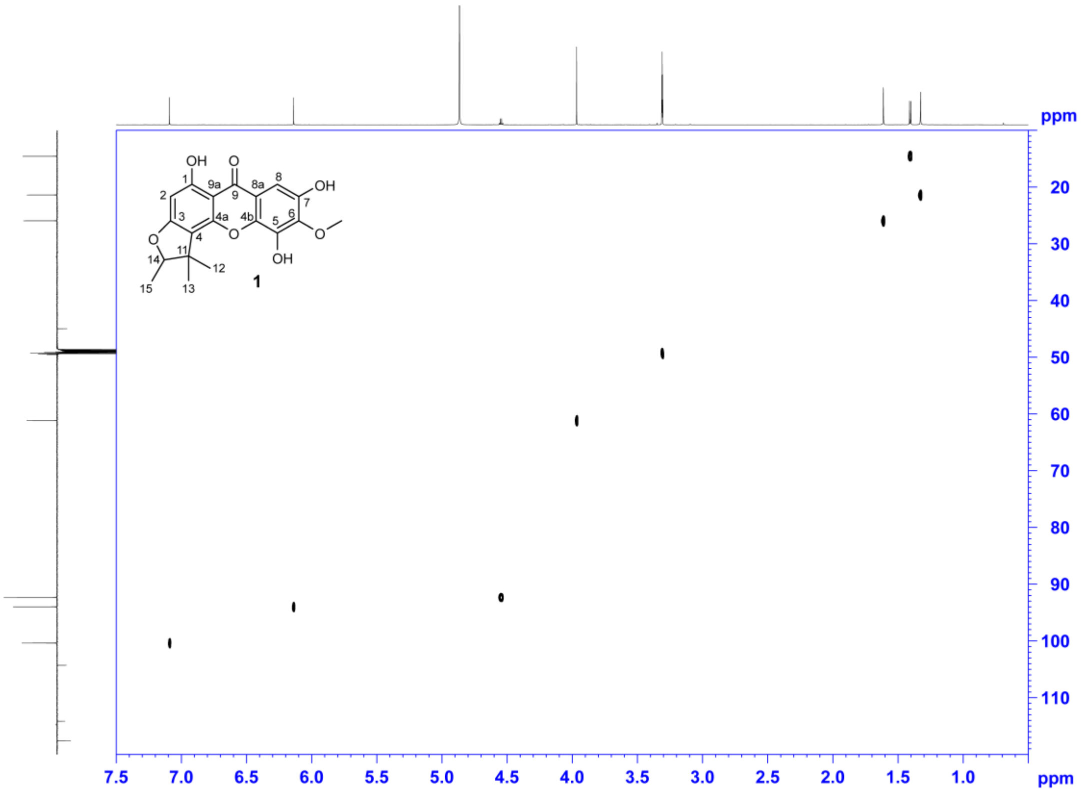

Fig.S11: HSQC spectrum of compound **1** (CD_3OD)

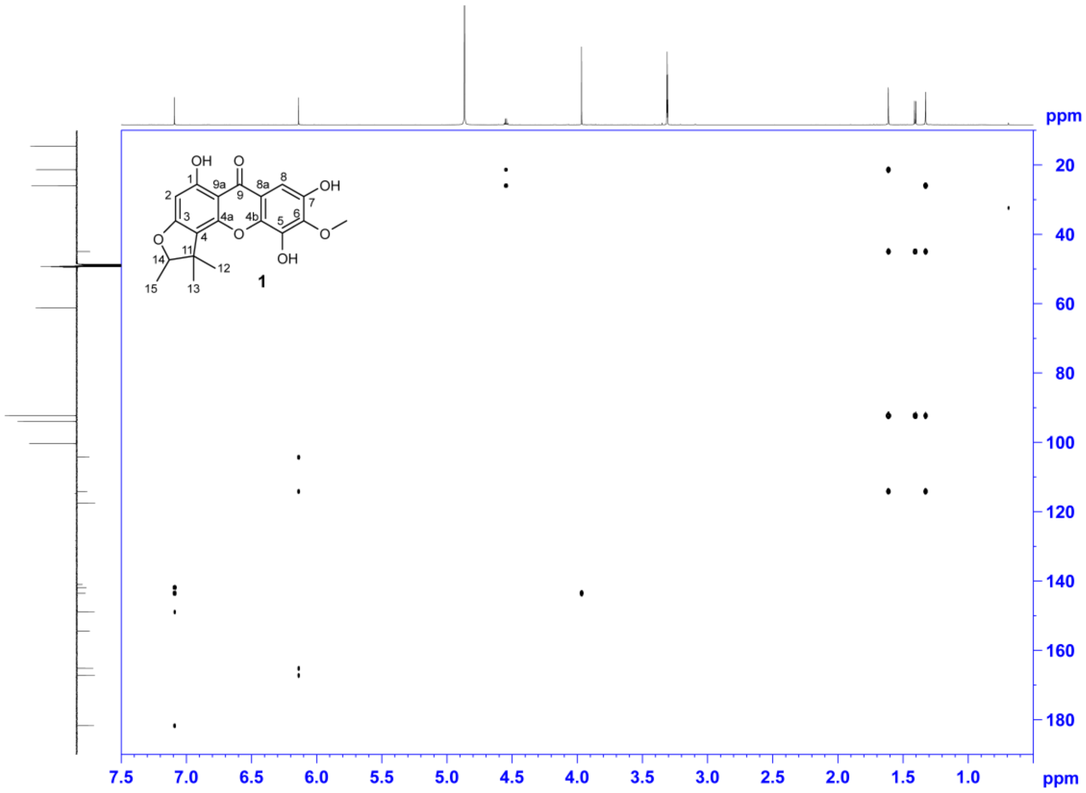

Fig.S12: HMBC spectrum of compound **1** (CD_3OD)

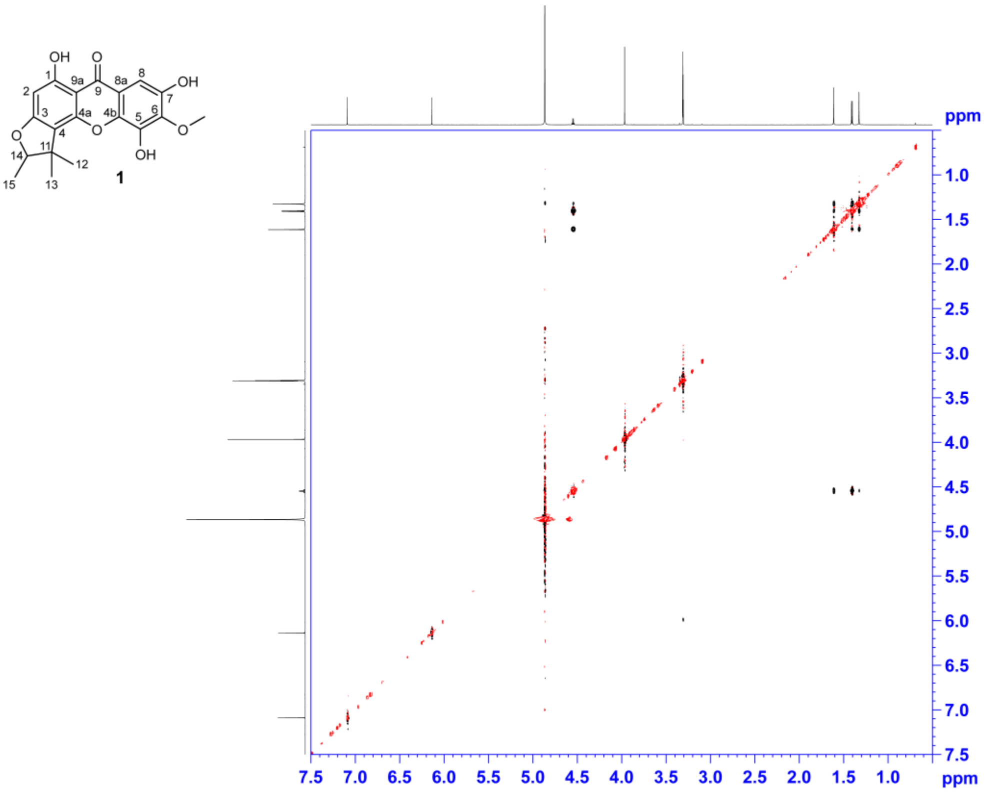

Fig.S13: NOESY spectrum of compound **1** (CD_3OD)

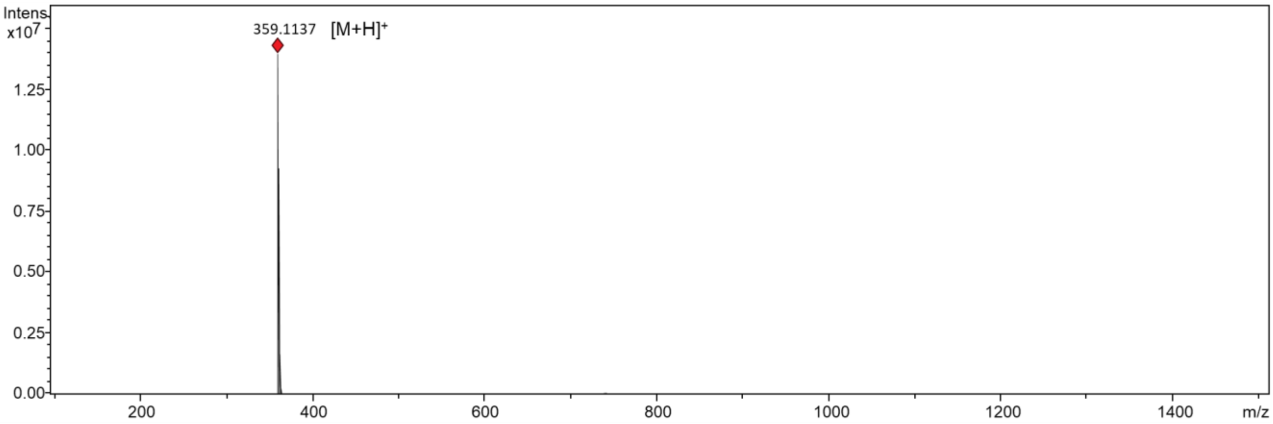

Fig.S14: MS1 spectrum of compound **1** showing the adduct $[M+H]^+$

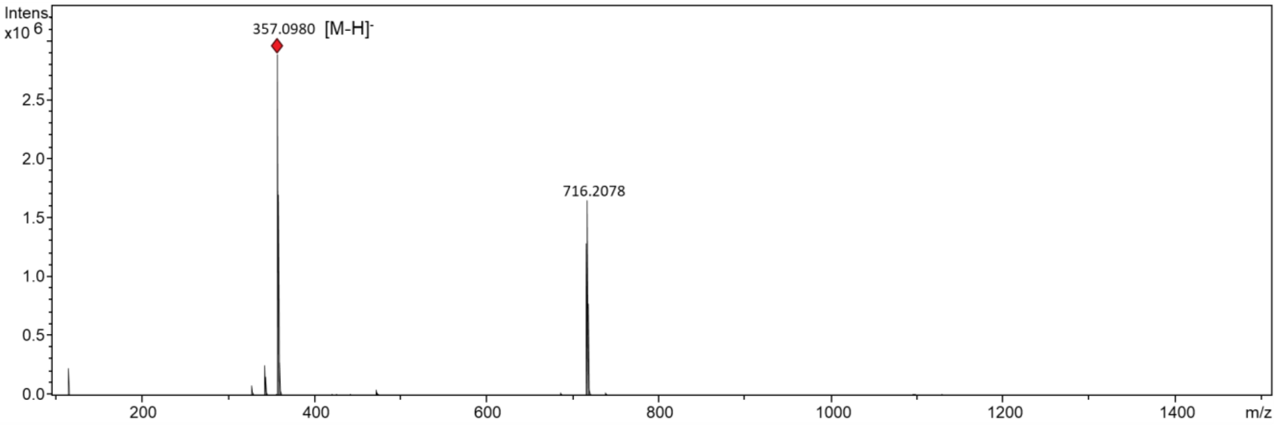

Fig.S15: MS1 spectrum of compound **1** showing the adduct $[M-H]^{-}$

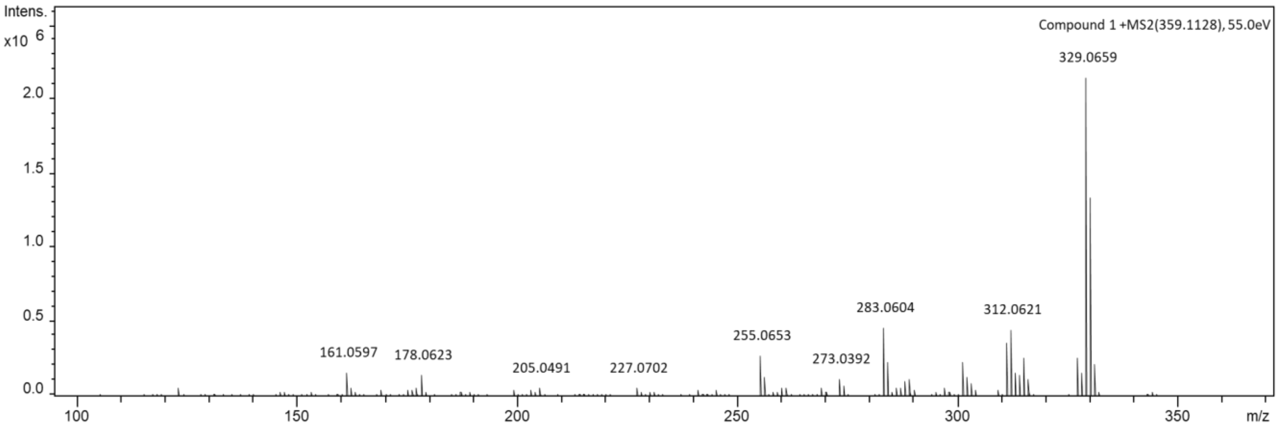

Fig.S16: MS2 spectrum of compound **1** fragmented using HCD at 55.0eV

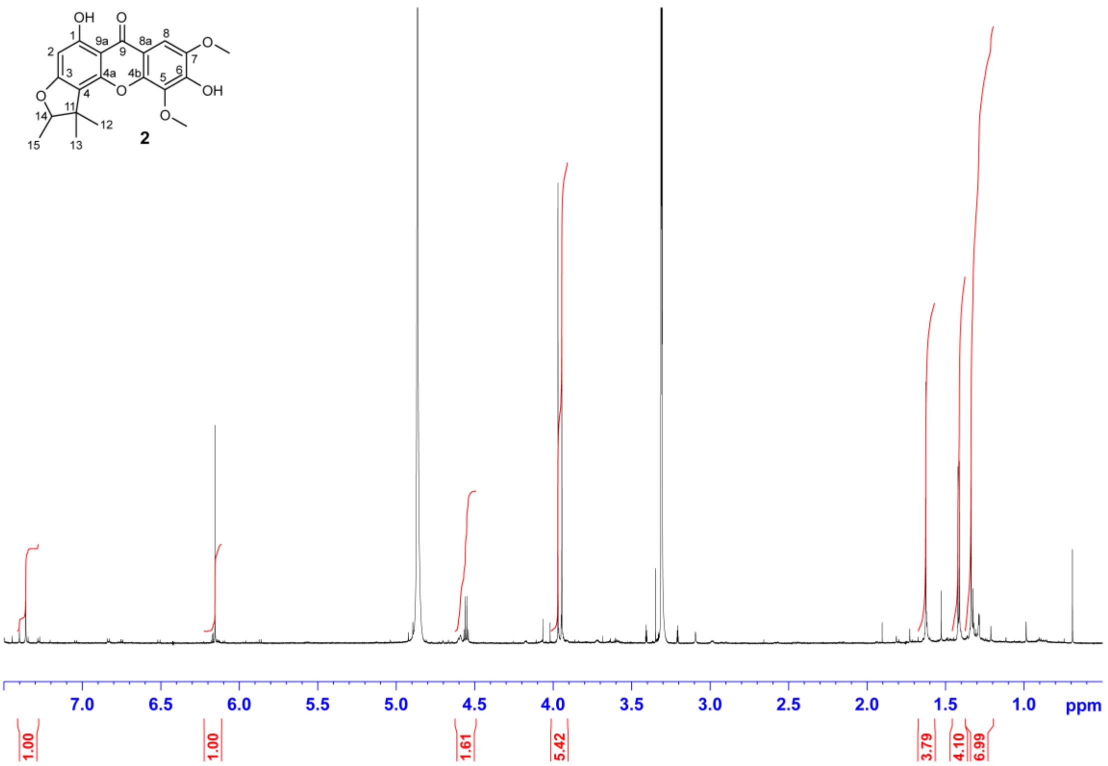

Fig.S17: ^{1}H NMR spectrum of compound **2** (CD_3OD, 700.40 MHz)

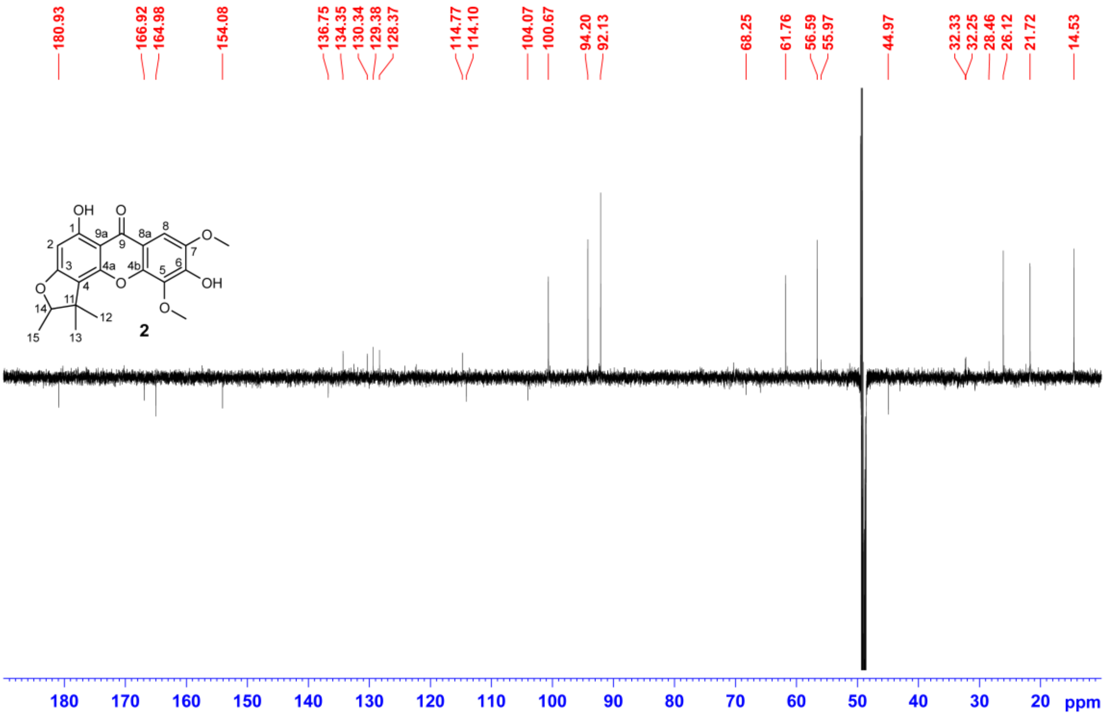

Fig.S18: ^{13}C NMR spectrum of compound **2** (CD_3OD, 176.12 MHz)

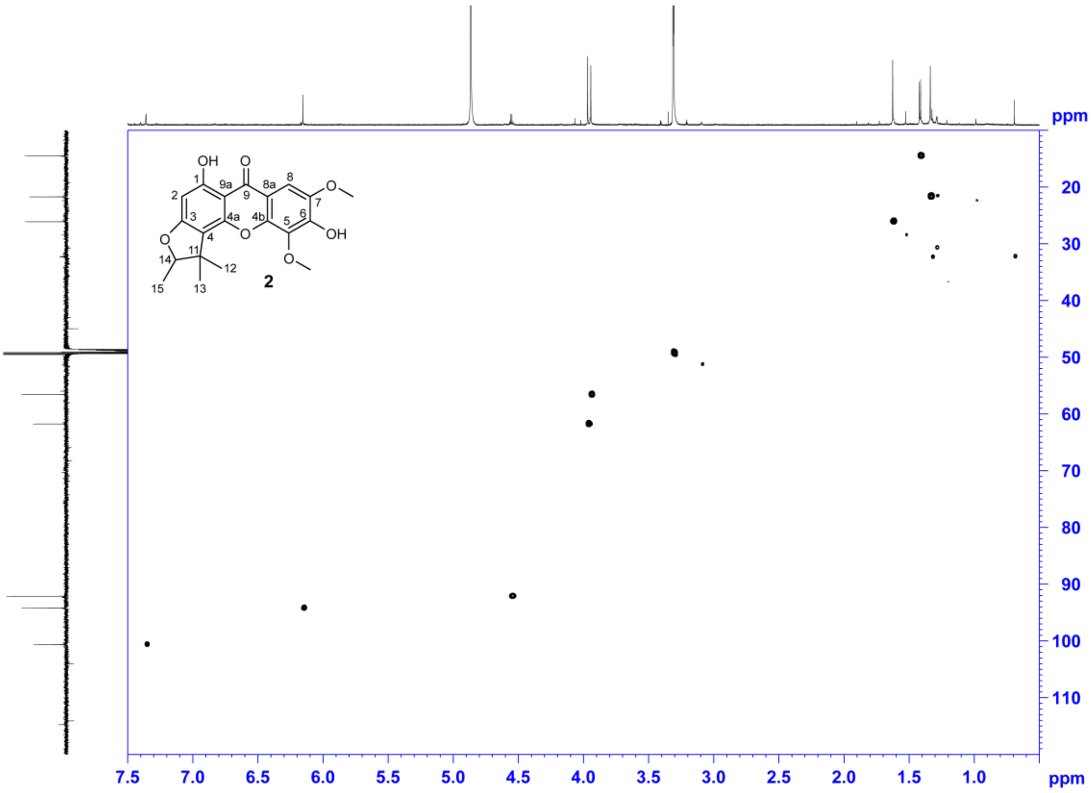

Fig.S19: HSQC spectrum of compound **2** (CD_3OD)

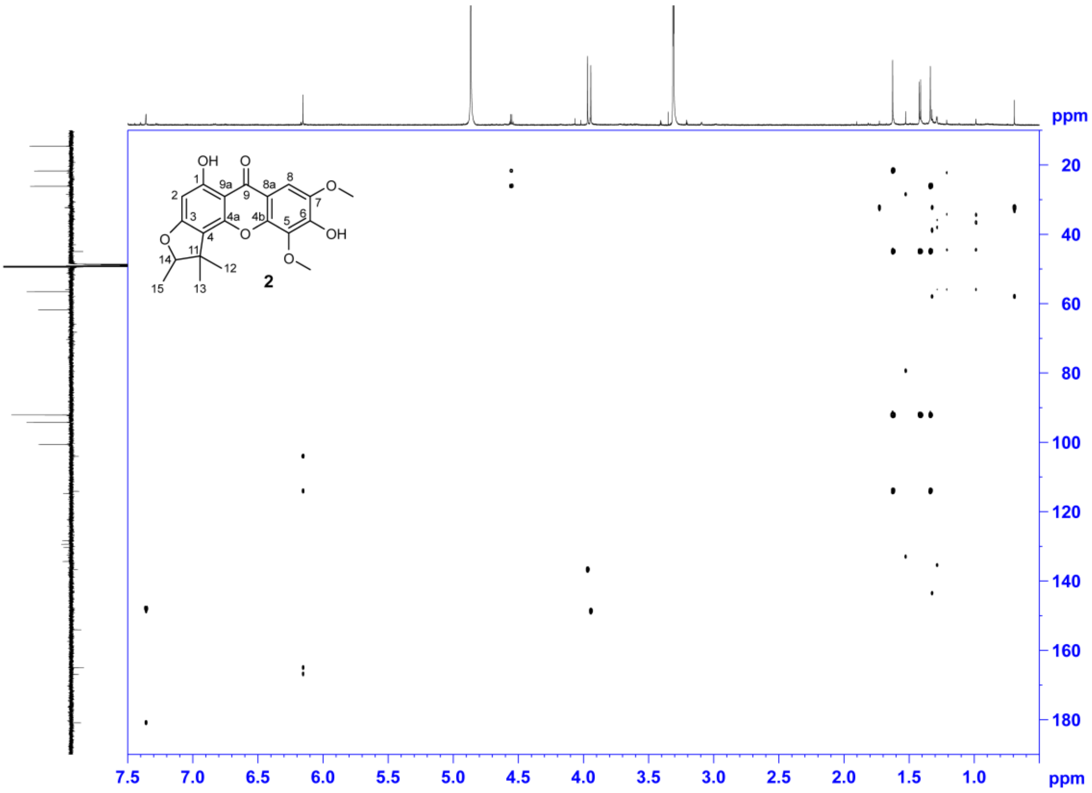

Fig.S20: HMBC spectrum of compound **2** (CD_3OD)

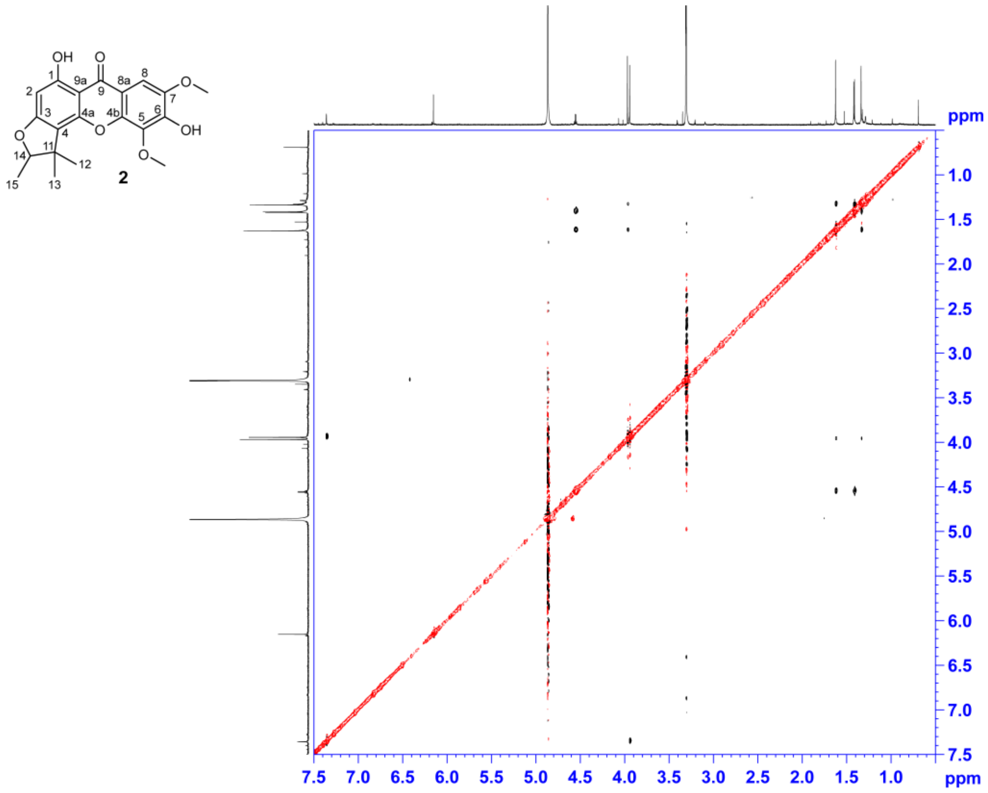

Fig.S21: NOESY spectrum of compound **2** (CD_3OD)

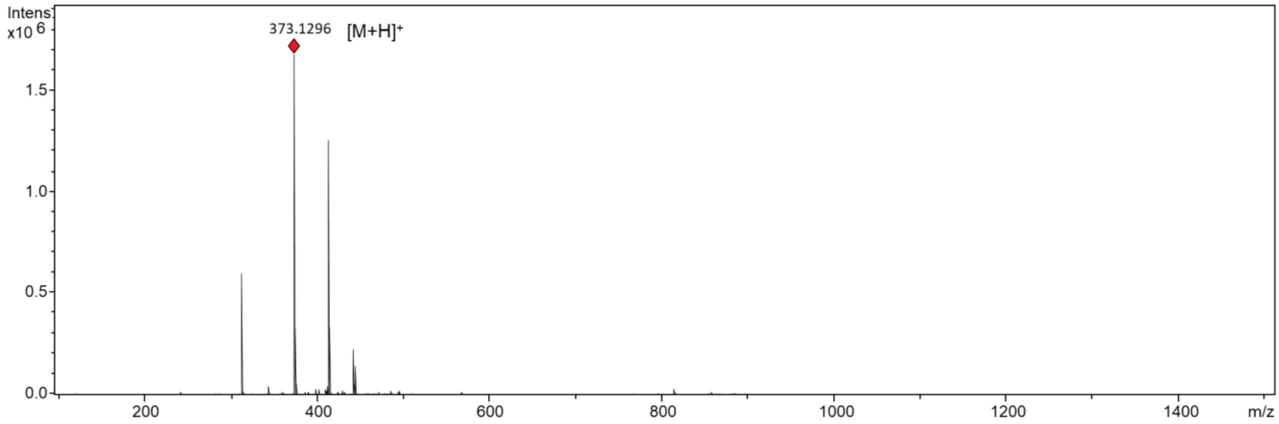

Fig.S22: MS1 spectrum of compound **2** showing the adduct $[M+H]^+$

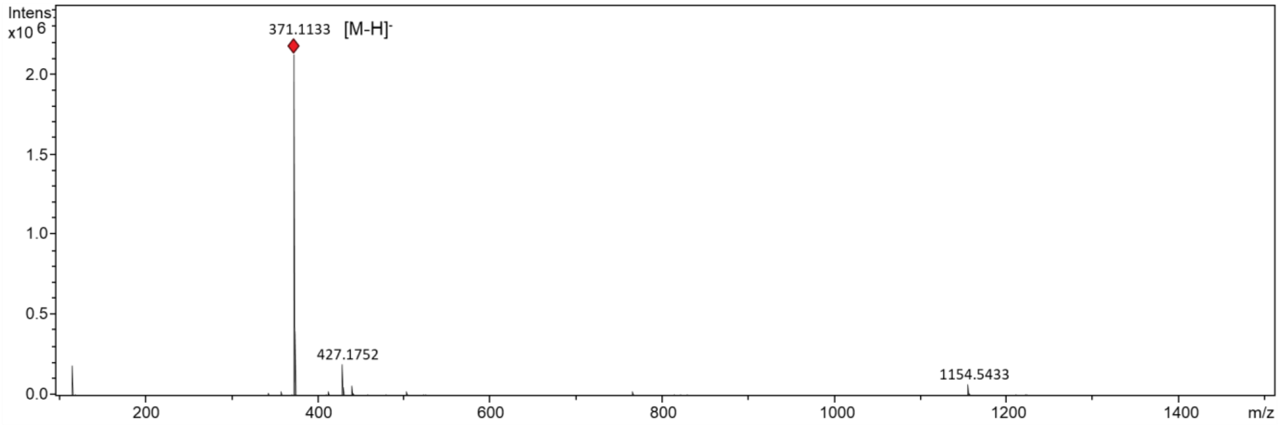

Fig.S23: MS1 spectrum of compound **2** showing the adduct $[M-H]^-$

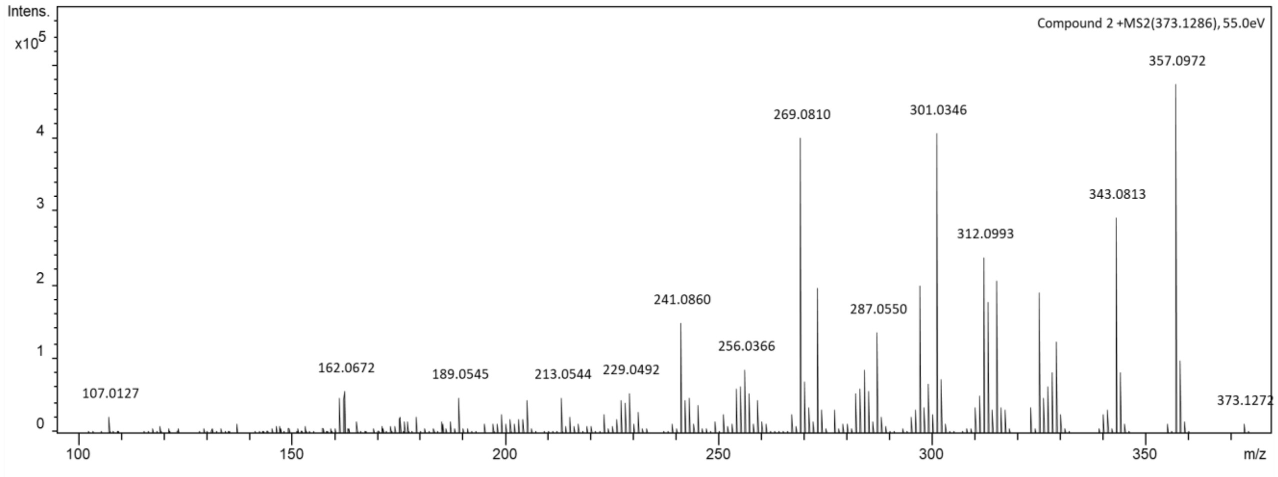

Fig.S24: MS2 spectrum of compound **2** fragmented using HCD at 55.0eV

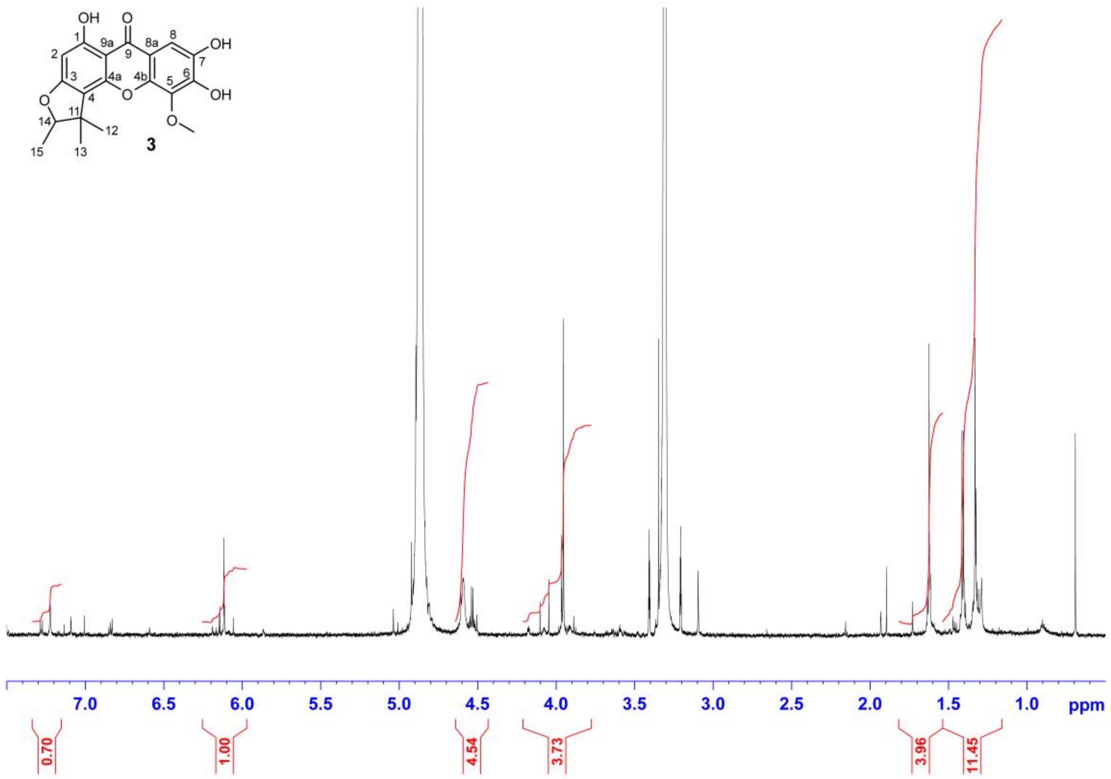

Fig.S25: ^{1}H NMR spectrum of compound **3** (CD_3OD, 700.40 MHz)

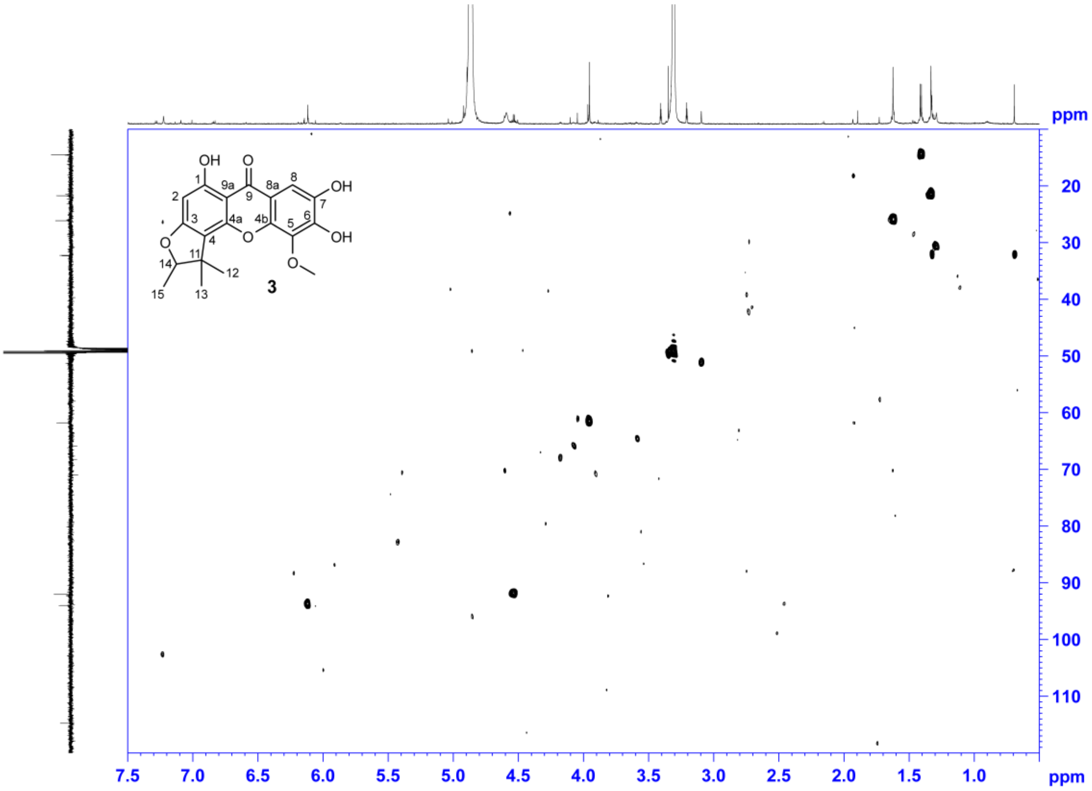

Fig.S26: HSQC spectrum of compound **3** (CD_3OD)

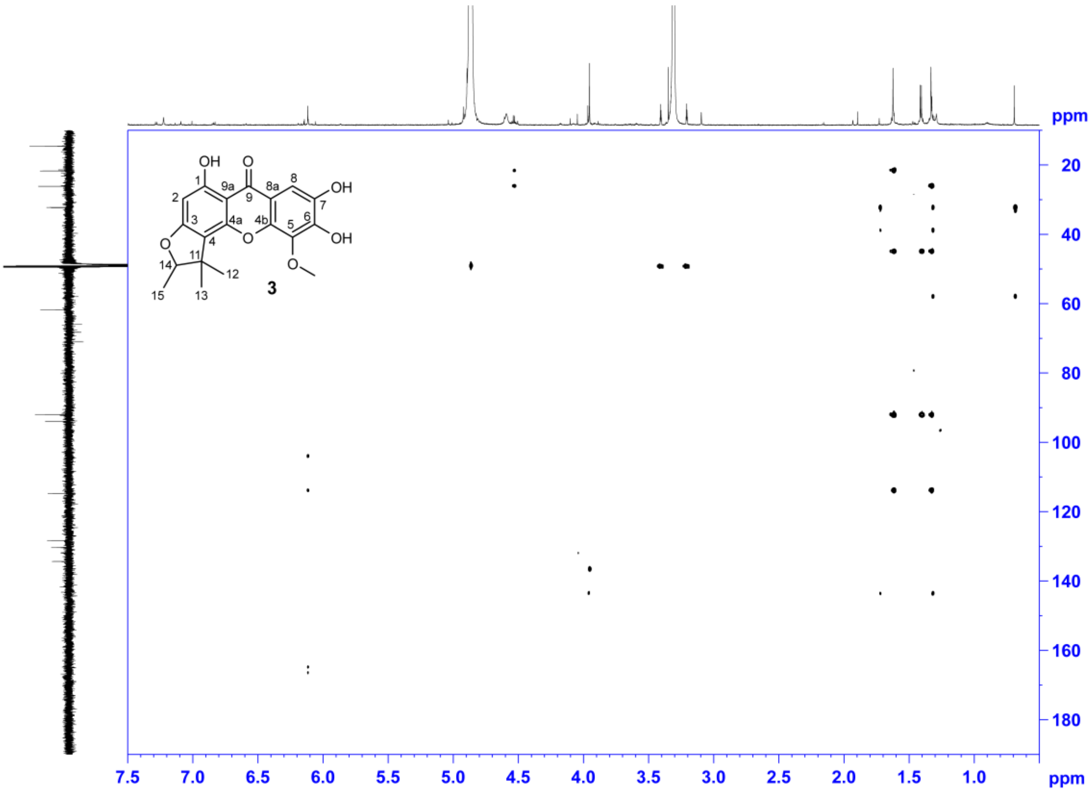

Fig.S27: HMBC spectrum of compound **3** (CD_3OD)

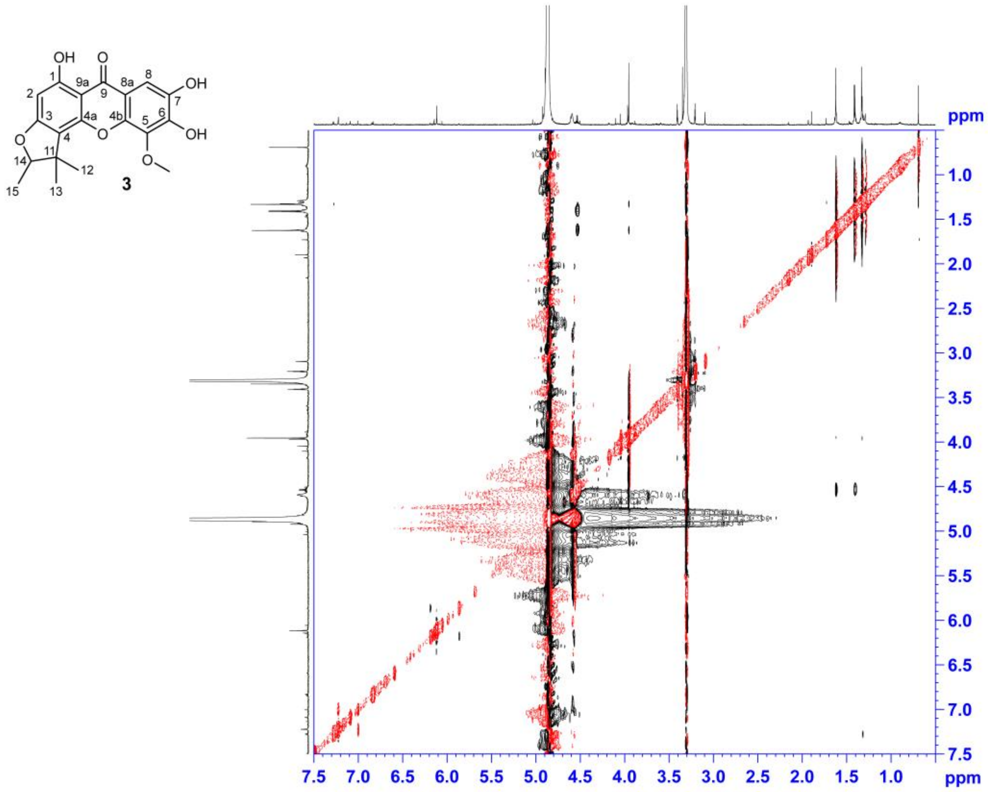

Fig.S28: NOESY spectrum of compound **3** (CD_3OD)

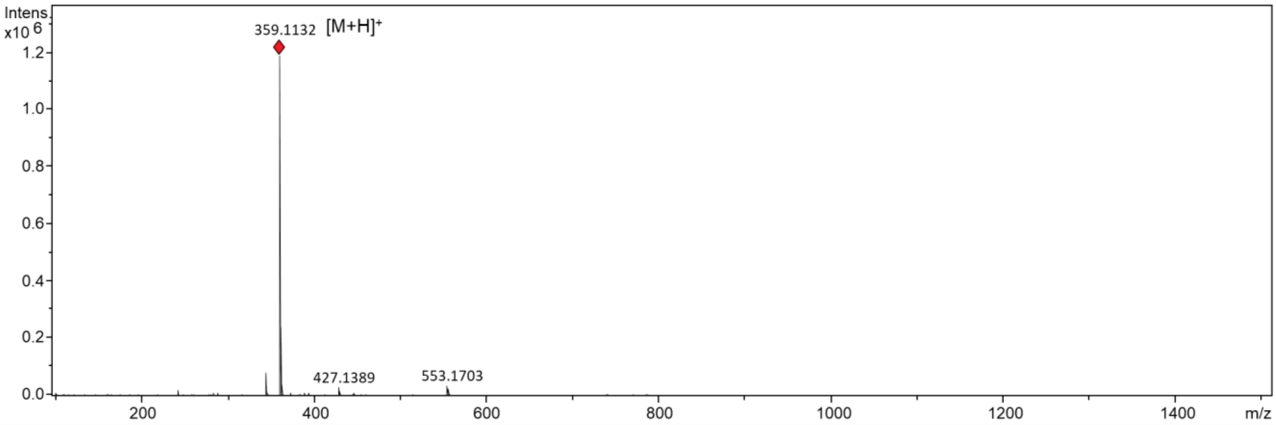

Fig.S29: MS1 spectrum of compound **3** showing the adduct $[M+H]^+$

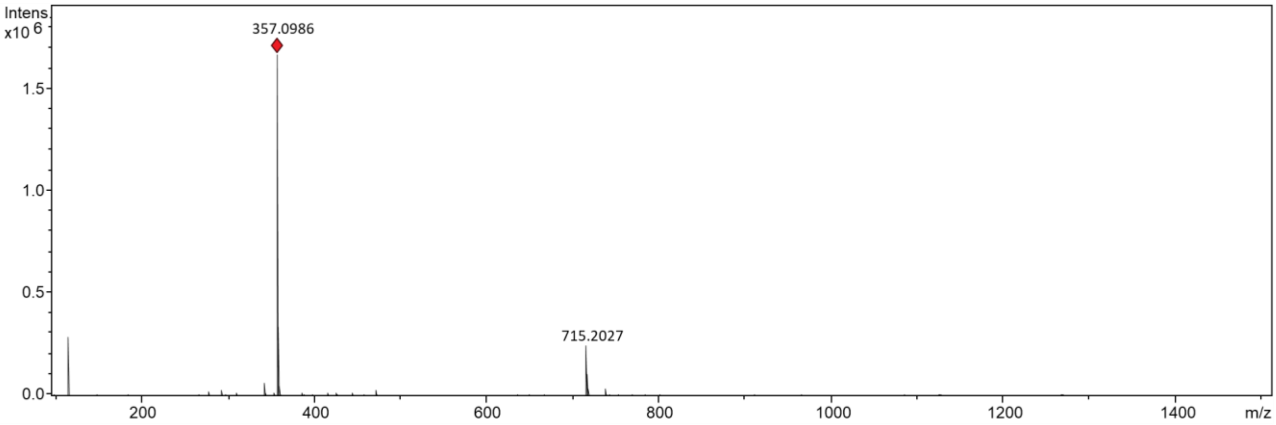

Fig.S30: MS1 spectrum of compound **3** showing the adduct $[M-H]^-$

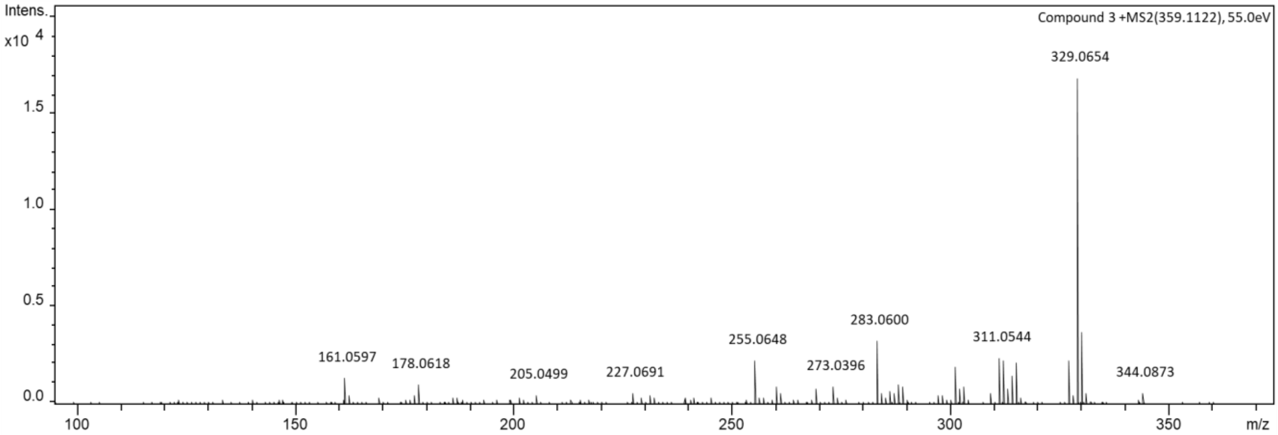

Fig.S31: MS2 spectrum of compound **3** fragmented using HCD at 55.0eV
